# What’s in a Name? Drug Nomenclature and Medicinal Chemistry Trends using INN Publications

**DOI:** 10.1021/acs.jmedchem.1c00181

**Published:** 2021-04-13

**Authors:** Marta Serafini, Sarah Cargnin, Alberto Massarotti, Gian Cesare Tron, Tracey Pirali, Armando A. Genazzani

**Affiliations:** Department of Pharmaceutical Sciences, Università del Piemonte Orientale, Largo Donegani 2, 28100 Novara, Italy

## Abstract

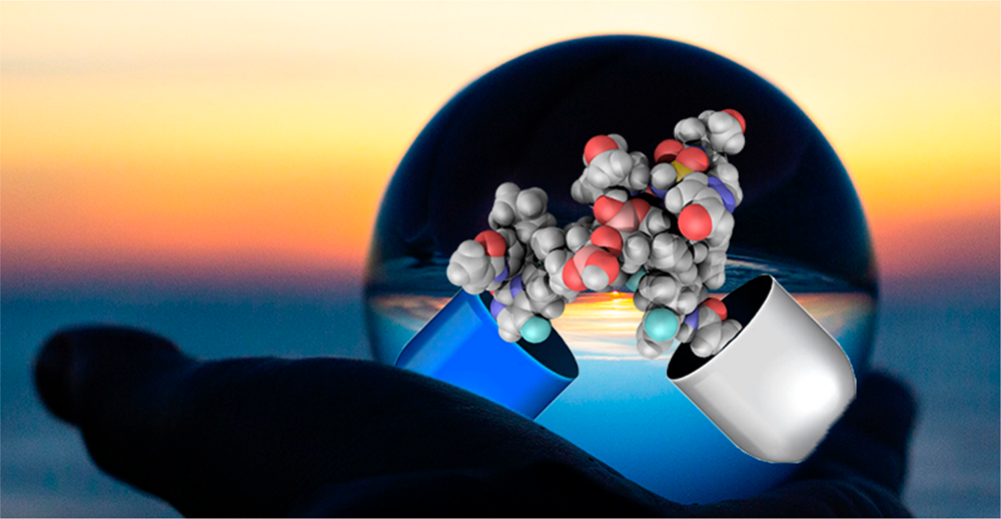

The World Health Organization assigns international nonproprietary names (INN), also known as common names, to compounds upon request from drug developers. Structures of INNs are publicly available and represent a source, albeit underused, to understand trends in drug research and development. Here, we explain how a common drug name is composed and analyze chemical entities from 2000 to 2021. In the analysis, we describe some changes that intertwine chemical structure, newer therapeutic targets (e.g., kinases), including a significant increase in the use of fluorine and of heterocycles, and some other evolutionary modifications, such as the progressive increase in molecular weight. Alongside these, small signs of change can be spotted, such as the rise in spirocyclic scaffolds and small rings and the emergence of unconventional structural moieties that might forecast the future to come.

## Introduction

Remember the biblical story of the Tower of Babel, in which the attempt to build a tower that would reach the heavens by the Babylonians was disrupted by the inability of the builders, that spoke different languages, to understand each other? Well, apparently there are today over 7000 spoken languages, and 34 of them are spoken by at least 45 million people.^1^ If each pharmacological active principle were to be called differently in each country and language, it would be disastrous for health as well as for scientific progress. Think, for example, of pharmacovigilance, of scientific publications, and of those traveling around the world.

Brand names that exist in most parts of the world well-exemplify this situation. In many countries the same drug is sold with multiple brand names, and, to make matters worse, these brand names change from country to country. While chemists might think that International Union of Pure and Applied Chemistry (IUPAC) names represent unique identifiers, it must be acknowledged that these are complicated, almost impossible to memorize for the lay public, and error prone. A common name, short and easy to pronounce, that identifies the same medicine everywhere in the world, is therefore required.

This was realized soon after World War II, and in 1953 the World Health Assembly, the governing body of the World Health Organization (WHO), passed resolution WHA3.11, which stated that an expert Committee of the WHO “*should undertake the selection and approval of non-proprietary names for drugs*” together with the recommendation that national pharmacopoeias should adopt such names.^2^ Such a resolution was the birth of the international nonproprietary names (INN) system for drug identification that we still use today for the effective and safe identification of medicines, for safe prescribing, and for teaching. It has also been a pivotal pillar for the development of the generic market of off-patent drugs, whereby in most countries the INN substitutes the brand name and allows therefore to unlink the manufacturer from the therapeutic effect. The INN has not substituted naming agencies altogether (e.g., in the United States (U.S.), England, Japan, and China, to cite some, national naming agencies are still active), but intense cooperation between these agencies has led, with very few exceptions, to identical names around the world. To imagine a world in which drugs are identified with different names, think if all drugs used were in the situation of paracetamol, salbutamol, and adrenaline, which in the U.S. are known, respectively, as acetaminophen, albuterol, and epinephrine.^3,4^ Fortunately, these are some of the very few exceptions.

While the present review will concentrate on the INN, it is interesting that contiguous fields have also adopted common names for their products, as a question of safety or consumer protection. Cosmetic ingredients, for example, have common International Nomenclature Cosmetic Ingredient (INCI) names issued by the Personal Care Product Council.^5^ Pesticides and agricultural chemicals also have their standardization for naming, which is the result of several different naming committees. In the U.S., the American National Standards Institute (ANSI) issues common names for chemicals, although other agencies, such as the International Standards Organization (ISO), or national standard organizations (e.g., British) also do so. It may occur that a few molecules receive common names by multiple bodies for different uses, and, while in most instances these names coincide (e.g., ascorbic acid is both an INCI and an INN), this is not always the case (e.g., nicoboxil is an INN, while the corresponding INCI is butoxyethyl nicotinate; the INN cetomacrogol 1000 corresponds to the INCI ceteth-20, both identifying the same tensioactive molecule; the ISO insecticide trichlorfon is equivalent to the INN drug metrifonate, once used for schistosomiasis,^6^ and the INN oxindanac, an anti-inflammatory drug that has never reached the market, is equivalent to the ISO herbicide quinclorac).

### How are INNs Defined?

INNs are issued upon request to the WHO and, in particular, to the Secretariat of the Expert Advisory Panel of the International Pharmacopoeia and Pharmaceutical Preparations, designated for this purpose, also known as the “INN Expert Group”. The INN Expert Group is composed of experts from all over the world that are led and coordinated by a Secretariat. Experts may include, among others, medicinal chemists, pharmacologists, biochemists, molecular biologists, and clinicians. Given that experts change over time and the scientific community modifies viewpoints on what is important in a drug, the composition of the group may also have an impact on the names chosen for a particular substance, with more or less emphasis given to the chemistry/structure, to the nature of the active principle, or to the mechanism of action. The current composition may be found on the WHO Web site.^7^ To provide consistency across fields, the meetings of the Committee are also attended by a number of other learned scientists, for example, representatives of national naming agencies and pharmacopeias, IUPAC experts, and Anatomical Therapeutic Chemical Classification System (ATC) code experts.

The person or company requesting the INN may propose a name that should abide by the rules and conventions of the INN system, and this may be accepted or modified by the Committee.^8^ Briefly, the core element of the INN is the stem, which is composed of one or two syllables, and is usually located at the end of the drug name. Such a stem identifies drugs that have a shared feature, usually the mechanism of action, although it can also be a therapeutic use or a chemical/structural characteristic. As an example, think of proton pump inhibitors: ome*prazole*, esome*prazole*, panto*prazole*, rabe*prazole* all share the common stem *prazole* that identifies “antiulcer, benzimidazole derivatives” (these brief definitions are given by the Committee once a stem is officially identified).^9^ If drugs are not recognized by the INN Expert Group as being part of a broader category, they will be assigned a unique ending. If other molecules that share the new feature will have an INN requested for them in the future, then these will receive the same ending, and the committee will promote it as an official stem. The WHO publishes a “stem book” and regular updates that can be freely consulted.^9^ Yet, it is not only the stem that qualifies a name, as there are prefixes (a syllable at the beginning of the INN), infixes (a syllable in the middle of the word), or suffixes (a syllable at the end of the INN) that characterize the name and may give information to the learned reader. For example, the syllable -*gli*- characterizes many drugs used in diabetes (e.g., *gli*benclamide, cana*gli*flozin, sita*gli*ptin, saro*gli*tazar, rosi*gli*tazone; note that *-glifozin*, *-gliptin*, *-glitazar*, and -*glitazone* are all stems identifying different drug classes and that the prefix *gli-* identifies anti-hyperglycemics, which are sulfonamide derivatives; for examples of the most frequent stems used by the INN Expert Group since 2000, see Table 1 below. Another example of how INNs work is represented by the manner by which chiral switches are represented in INNs, in which an infix illustrates the change, for example, in *es*omeprazole (S isomer of omeprazole), *es*citalopram (S isomer of citalopram), *es*ketamine (S isomer of ketamine), *levo*floxacin (levo-rotatory stereoisomer of ofloxacin) or *dex*ketoprofen (the dextro-rotatory stereoisomer of ketoprofen).

**Table 1:** Stems of SCEs Used at Least 10 Times in the Last 20 Years

ranking of stems in SCEs	stem	definition	example
#1^a^	-*ine*	alkaloids and organic bases	atrop*ine* (according to WHO, ∼17.5% of INNs have used this stem in lists p1-p119)^9^
#2^b^	-*tinib*	tyrosine kinase inhibitors	ima*tinib*
#3^b^	-*stat*–/–*stat*	enzyme inhibitors	atorva*stat*in, cobici*stat*
#3^b^	*vir*–/–*vir*–/–*vir*	antivirals (undefined group)	mara*vir*oc, remdesi*vir*
#5^b^	*gli*–/–*gli*-	antihyperglycaemics	*gli*benclamide, dapa*gli*flozin
#5^a^	-*one*	ketones	nalox*one* (according to WHO, ∼7.5% of INNs have used this stem in lists p1-p105)^9^
#7^b^	-*imod*	immunomodulators, both stimulant/suppressive and stimulant	fingol*imod*
#8	-*sertib*	serine/threonine kinase inhibitors	no drug is yet on the market
#9^c^	*fos*–/–*fos*-	phosphorus derivatives; various pharmacological categories belonging to “*fos*”, other than insecticides, anthelminthics, pesticides, etc.	*fos*carnet, so*fos*buvir
#10	-*lisib*	phosphatidylinositol 3-kinase inhibitors, antineoplastics	idela*lisib*
#11^b^	-*ast*	antiallergic or anti-inflammatory, not acting as antihistaminics	zafirluk*ast*
#11^b^	-*tant*	neurokinin (tachykinin) receptor antagonists	aprepi*tant*
#13	-*anib*	angiogenesis inhibitors	ninted*anib*
#13	-*bulin*	antineoplastics; mitotic inhibitors, tubulin binders	eri*bulin*
#13	-*ciclib*	cyclin dependent kinase inhibitors	palbo*ciclib*
#16^b^	-*abine*	arabinofuranosyl derivatives; nucleosides antiviral or antineoplastic agents, cytarabine or azacitidine derivatives	capecit*abine*
#17	-*entan*	endothelin receptor antagonists	bos*entan*
#17	-*oxacin*	antibacterials, nalidixic acid derivatives	levofl*oxacin*
#19	-*conazole*	systemic antifungal agents, miconazole derivatives	keto*conazole*
#19^b^	*prost*–/–*prost*–/–*prost*	prostaglandins	*prost*alene (veterinary use), miso*prost*ol, latano*prost*
#21	*-caftor*	cystic fibrosis transmembrane regulator (CFTR) protein modulators, correctors, and amplifiers	iva*caftor*
#21	-*nicline*	nicotinic acetylcholine receptor partial agonists/agonists	vare*nicline*
#23	-*tecan*	antineoplastics, topoisomerase I inhibitors	irino*tecan*
#23	-*adol*–/–*adol*	analgesics	tram*adol*
#23	-*ciguat*	guanylate cyclase activators and stimulators	rio*ciguat*
#23	-*denoson*	adenosine A receptor agonists	rega*denoson*
#27	-*parib*	poly-ADP-Ribose polymerase inhibitors	ola*parib*
#27	-*terol*	bronchodilators, phenethylamine derivatives	formo*terol*
#27	-*vaptan*	vasopressin receptor antagonists	tol*vaptan*

a A number of other stems include this one (e.g., -*terone* for androgens).

b These stems possess substems that categorize in more detail. For example, -*brutinib*, -*citinib*, -*ertinib*, and -*metinib* group together tyrosine kinase inhibitors (-*tinib*) with the same target (Bruton kinase, Janus Kinase, EGFR, MAPK, respectively).

c The stem -*fos* is, instead, used for insecticides, anthelmintics, pesticides, etc., phosphorus derivatives.

In general, INNs are selected for the active moiety of drugs. Pharmaceutical reasons may drive slight modifications to be made during the drug development process or during the lifetime of the product. This is the case of salified forms, ester prodrugs, hydrates or solvate forms, combination products, or complexes. To reduce the number of published INNs, all these cases do not result in the publication of a different INN from that assigned to the active moiety but to the creation of a modified INN (INNM), which does not necessarily need to be devised by the INN Expert Group and may be created by the manufacturer. Nonetheless, INNMs follow strict rules and lead to two- or three-word names, the first of which refers to the active principle, and the subsequent ones are attributable to the inactive moiety. Yet, in some specific cases, the radicals or groups composing the inactive parts are highly complex, and therefore the INN Expert Group selects a common name for them, and WHO publishes periodically a list of “names for radicals and groups” to be used in combination with an already assigned INN.^10^

For those drugs that allow it, and that have emerged more recently on the horizon, more rigid schemes have been devised to name substances. This is particularly true for monoclonal antibodies and for advanced gene and cell therapies. The naming of these substances has recently been reviewed elsewhere.^11^

It has been advocated that INNs, stems, and radicals could be an excellent learning tool for students.

**Figure 1:**
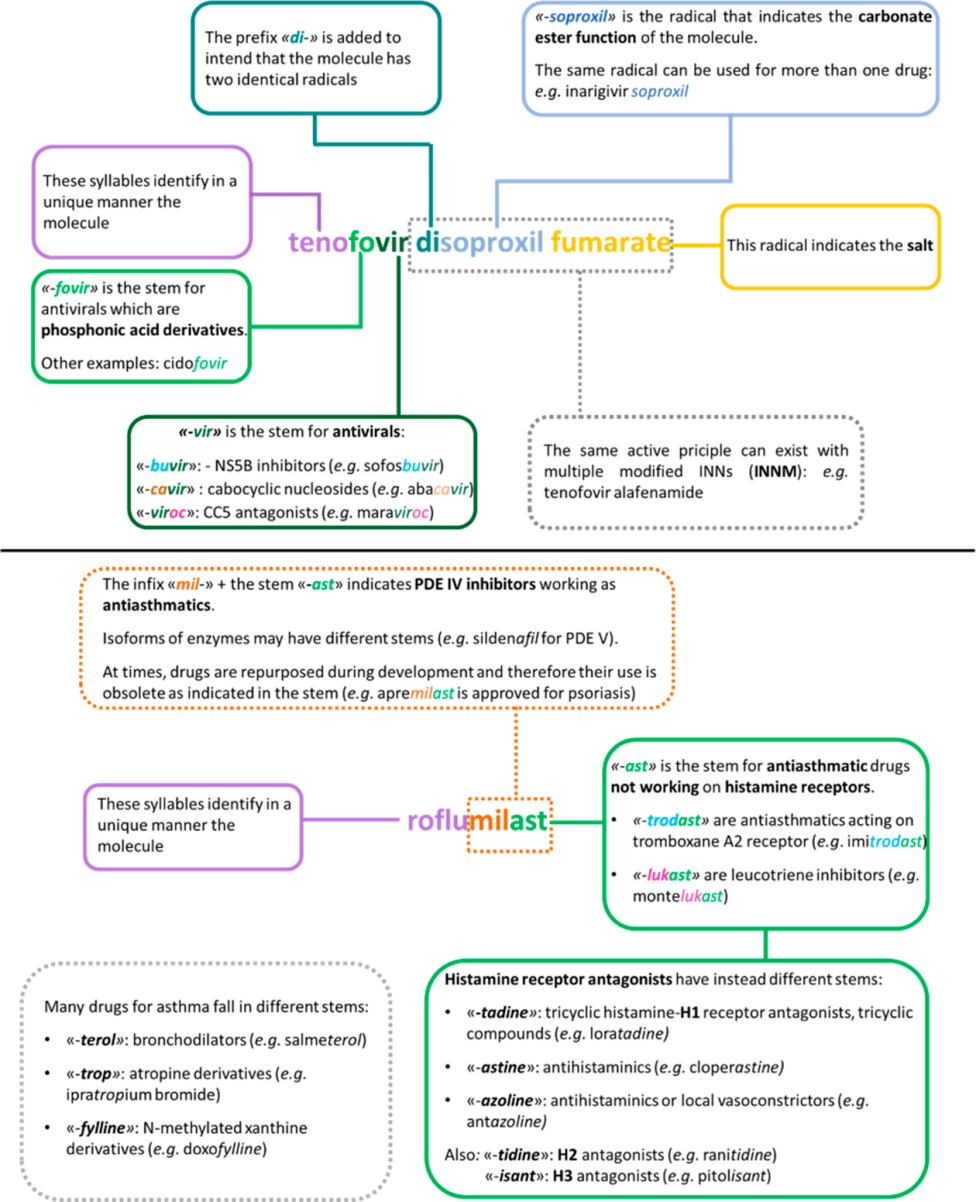
Use of stems, prefixes, infixes, and suffixes in INNs. The definitions of the stems in the figure are the official definitions that can be found in the stem book.^9^

Figure 1 illustrates part of the value of this approach. Starting from a name, links can be made to other drugs of the same class, to drugs that share the same mechanism of action, to drugs that have similar structural features, to drugs that have the same therapeutic use, and to drugs that have been salified or esterified in similar manners, thus creating mnemonic aids. The INN Expert Committee recently also set up a School of INN to educate on how to construct, design, and interpret INNs.^12^

The name chosen by the Committee, which meets in Geneva twice a year, is known as the proposed INN (pINN) and is circulated among all stakeholders (Ministries of Health, industries, learned societies, etc.) that may object to the name for a number of reasons, including trademark infringements, similarity to other substances, or inappropriate meaning in a particular language. If an objection is raised, then the Committee is asked to re-evaluate the name, while if no objections are received by WHO, the name is, approximately a year later, declared a recommended INN (rINN). Each year two lists of the pINNs are published on the WHO Web site^13^ reflecting the choices made by the Committee. At present, list p123 has been published, and the first list dates to 1953. The latest rINN list published in 2020 is r84, and the difference of numeration between the proposed and the recommended list dates back to the initial years, in which several pINN lists were usually incorporated in the same rINN list. Since 2000, the two lists differ minimally, although some substances may see a deferral of their publication because of objections.

Regulatory agencies (e.g., European Medicines Agency (EMA), Food and Drug Administration (FDA), Pharmaceuticals and Medical Devices Agency (PMDA)) require that drugs submitted for their assessment are identified with an INN (or with a name given by the national naming agency), and therefore an INN is in most instances issued before the completion of clinical trials. While the WHO INN Expert Committee has a preference for drugs to be submitted after the beginning of Phase II trials, this is not always the norm, and an INN can be issued before or after, although it is very unlikely that applicants submit an application in the absence of encouraging data from Phase I trials. While receiving an INN for a drug increases the value of the portfolio, as it gives the feeling that it is closer to the market, companies are well-aware that the INN publication will allow competitors to know their intentions on the lead molecule being developed.

In our opinion, the pINN and rINN publications^13^ are a unique opportunity to scrutinize drug development as well as the recent trends in drug research and development (R&D) (as they precede marketing by a few years, see below), and, to our knowledge, this has been overlooked by scholars. Moreover, the INN publications have never been scrutinized from a medicinal chemistry viewpoint, unlike other chemical catalogues, such as FDA-approved drugs,^14−21^ including veterinary drugs,^22^ the Essential Medicines List (EML),^23^ or molecules published in medicinal chemistry journals.^24,25^ In the present contribution, we concentrated on the chemical entities published in the INN lists in this millennium. Indeed, the publication of the INN represents at times the first public disclosure of the molecule and, while obviously not all substances that have been assigned an INN turn into approved drugs, these are all compounds that the industry has invested in. It is therefore an intermediate approach between analyzing only successful molecules (approved by the FDA or present on the EML) and analyzing discovery compounds (e.g., molecules published in the *Journal of Medicinal Chemistry*), as it includes promising molecules, which might turn out to be successes or clinical failures. Our approach of concentrating on molecules in the last 20 years gives us the ability to investigate long-term trends in drug development and in medicinal chemistry, although we acknowledge that historical trends could be also investigated by taking all molecules published since 1953, or, on the contrary, features of newer molecules could be investigated by taking only the past few years.

### Bird’s-Eye View Analysis of the INNs of the Millennium

From 2000 to September of 2020, 3159 substances have received an INN published as a rINN. Note that r43 (2000) presents 43 substances whose publication had been delayed because of long-lasting objections and most likely date to the 1990s.^26^ For our analysis, we used drugs listed in the recommended lists from 2000 to 2020 (r43 to r83), and we added the drugs present in the latest two proposed lists (p122 and p123), to include the most recent applications, leading to a total of 3456 molecules.

**Figure 2:**
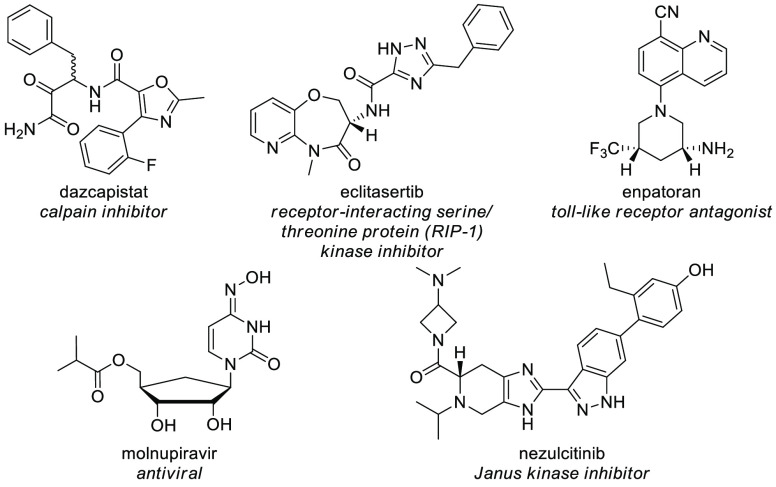
Structures of the five SCEs present in list p124 devoted to drugs potentially of use in the COVID pandemic.

Not surprisingly, 2021 brought a surge of molecules, for which INNs were sought urgently, to be developed for the coronovirus disease of 2019 (COVID-19), and an extraordinary list (p124-COVID) was created for these drugs. These 25 drugs were not considered in our analysis. p124 is composed of monoclonal antibodies (*N* = 10), followed by RNA-based approaches (*N* = 5), organic compounds (*N* = 6), biologicals (*N* = 3), and advanced therapies (*N* = 1). Figure 2 depicts the five novel small chemical entities (SCEs) against severe acute respiratory syndrome (SARS) coronavirus 2 (CoV-2) found in this list. It is important to highlight, in this context, that the potential activity is self-declared by the applicant, and it is not up to the Committee to evaluate data that back efficacy or safety claims.

As mentioned above, lists are published on the WHO Web site.^13^ Compounds are described to uniquely identify them (e.g., IUPAC, amino acid sequence), and the lists also include other information. In the proposed lists, organic compounds are also described by their brute formula, the Chemical Abstracts Service (CAS) number and the chemical structure as well as by the broad class they belong to, while recommended lists lack some information, such as the CAS number and drug class. The description of the class is usually broad and heterogeneous: for example, it may refer to its chemistry (e.g., vitamin D analogue), to its pharmacological action (e.g., antiviral), or to its mechanism of action (e.g., toll-like receptor antagonist), following a common law on how the first compounds of the class were first described.

At first, we visually inspected all 3456 molecules and classified them in broad categories (see definitions in ref (27)), which include “inorganic small chemical entities” (*N* = 2), “organic small chemical entities (SCEs)” (*N* = 2018), “biologicals” (*N* = 411), “monoclonal antibodies” (mAbs) (*N* = 578), “conjugates” (*N* = 83), “DNA/RNA-based therapies” (*N* = 91), “advanced therapies” (ATMPs) (*N* = 129), “polymers” (*N* = 25), “veterinary” (*N* = 45), and “mixtures” (*N* = 3). This classification required a number of compromises, and we do acknowledge that the great variety of compounds included means that some compounds could also have been classified differently. To represent this difficulty, 71 compounds were classified as “other”, as they did not meet our criteria of active principle (diagnostics, excipients, radiodiagnostics, sunscreens) or could not be reconducted to any other category (i.e., belzupacap sarotalocan).^28^

**Figure 3:**
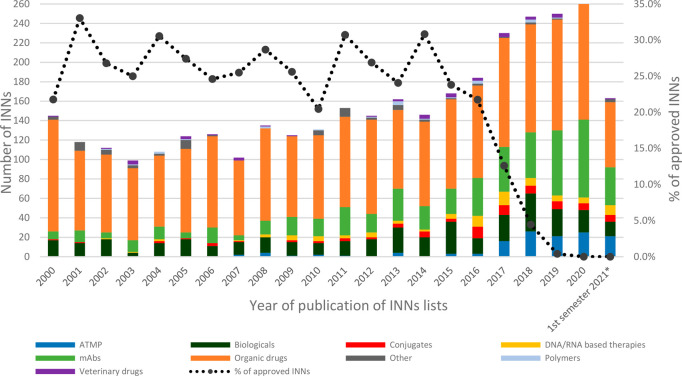
INNs issued between 2000 and 2021. Categories composed of less than 10 substances were excluded from the figure (two inorganics and three mixtures), while the same were included in the calculation of the percentage of approved INNs.

Figure 3 shows how these categories have changed over time, both from a quantitative and qualitative point of view. Notice that the number of applications over time has dramatically increased, starting from 40 to 60 INNs for each volume in the 2000s and rising to a maximum of 163 in the last published list (p123). The number of SCEs that have received yearly an INN has roughly remained the same (between 70 and 120 depending on the year), although their weight percentage-wise has decreased significantly (from ∼80% in volume r43 to ∼40% in the last volume), in favor of mAbs, biologicals, ATMPs, conjugates, and DNA/RNA therapies. It is often stated that SCEs are being replaced by biotechnological products, but the analysis made, instead, suggests that biotech compounds are added, and are not a substitute, of traditional medicinal chemistry molecules.

We then decided to evaluate how many of the molecules that are published in the rINN lists eventually become approved drugs in the main global markets, U.S., Europe, or Japan, and how anticipated is the disclosure in the INN publications. The dotted line in Figure 3 shows the percentage of drugs published by the WHO in that particular year eventually approved by the FDA,^29^ EMA,^30^ or PMDA.^31^ As it can be observed, between 20% and 30% of drugs from each list are approved. Data from 2017 onward are significantly lower, as most drugs from those years have not been approved yet, and this shows that the INN anticipates by a few years drug approval.

As a whole 639 of 3456 drugs (19%) were approved by at least one agency by Nov 1, 2020. Briefly, 493 (14%) were approved by the FDA, 441 (13%) by the EMA, and 345 (10%) by the PMDA. Notice that the database we used for PMDA includes approval data from 2004, and therefore the number of approved drugs in Japan is underestimated. INNs, by definition, are used globally, and it is possible that some drugs that have been classified as “not approved” in our analysis have been approved elsewhere in the world. To support this statement, we searched different Web sites and databases^32^ to investigate whether the molecules depicted in the figures of this review and not listed in the FDA, EMA, or PMDA sites had been authorized elsewhere in the world, and we found that a number of these are indeed marketed (mainly in South America or the Far East). This therefore suggests that approved drugs are underestimated, as our analysis does not take into account a number of other markets. When classified in broad categories, the percentage of approval for each class, considering U.S., Europe, and Japan, was found to be 24% for biologicals, 20% for SCEs, 15% for conjugates, 13% for mAbs, 12% for polymers, 10% for DNA/RNA based therapies, and 5% for ATMP, while no inorganic drug or molecule classified as a mixture was approved.

**Figure 4:**
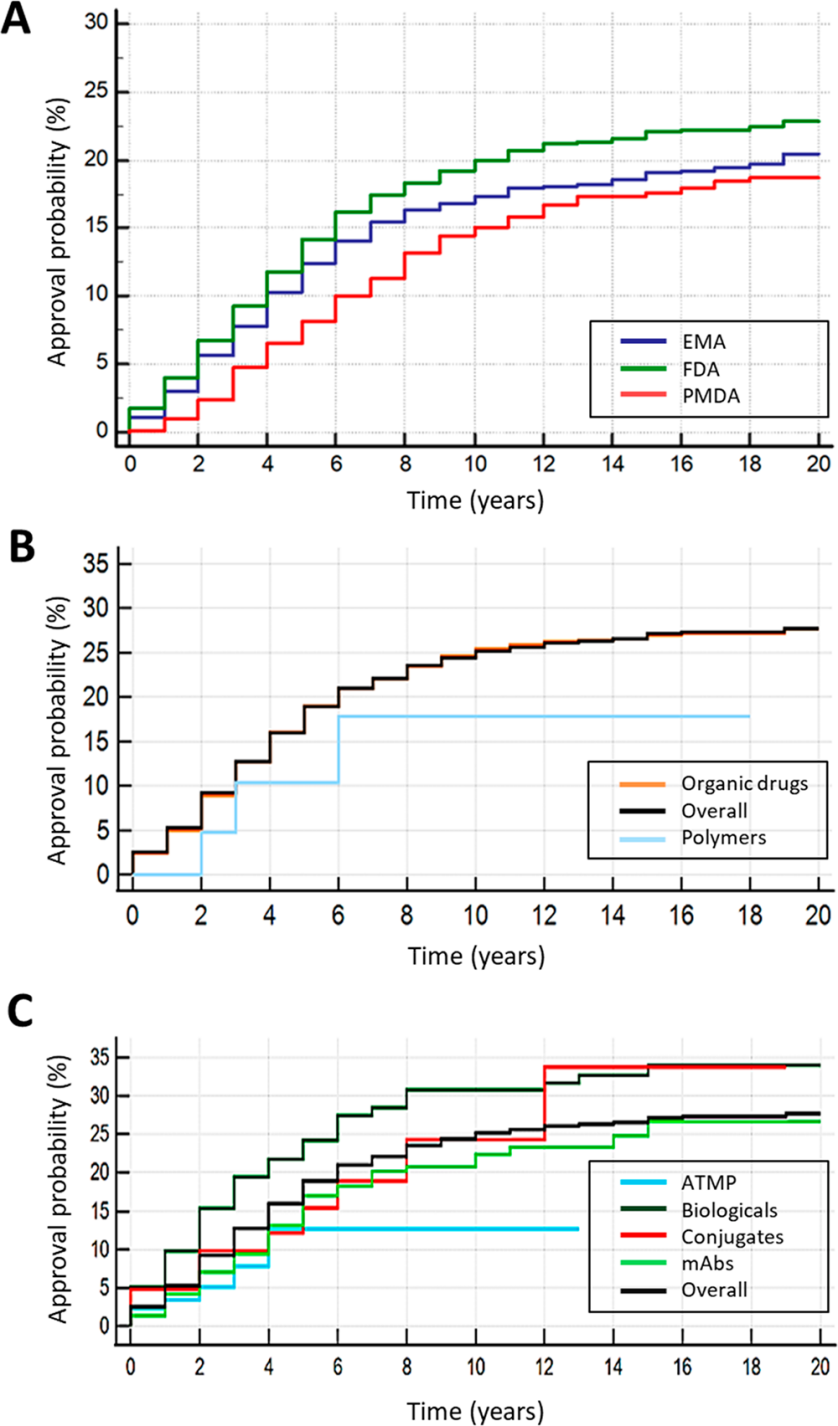
Probability of INN approval from the year of publication on the recommended INN list.

We then analyzed the time to approval in the three regulatory districts. This is displayed in the Kaplan–Meier plot in Figure 4. This manner of expressing data allows an estimation of events over time and, in this particular instance, the probability that an INN is approved after a given time. As it can be observed in panel A, it is estimated that ∼22.5% of the total INNs will be approved by the FDA, and a slightly lower number will be approved by the EMA and PMDA. It is estimated that half of these drugs will be approved within four years in the U.S. and Europe, while it will take slightly longer for Japanese approval (this latter analysis might be nonetheless skewed by the loss of data between 2000 and 2004). Very few drugs are approved after 10 or more years from the INN publication. Notice that our analysis is performed on the rINN list, but a disclosure by the WHO in the pINN lists occurs approximately a year earlier. In brief, therefore, it is expected that, for each new list published, a fifth to a quarter of the drugs will be authorized and that half of these authorizations will occur within the first five years. We also investigated whether the different categories of drugs took different amounts of time to get approved (Figure 4B,C). Briefly, the median approval time for ATMPs was two years; for polymers and biologics it was three years, for small chemical entities it was four years, for mAbs it was five years, and for conjugates it was six years.

## Analysis of the Medicinal Chemistry of the INNs in the Last 20 Years

Having characterized the data set, we then decided to proceed evaluating the medicinal chemistry solely of SCEs listed in the INN publications. While we took inspiration from other reviews on chemical catalogues for a systematic analysis,^14−22^ we also arbitrarily concentrated on particular aspects or molecules that caught our attention or on details that have recently emerged in the literature. The main goal of the review is to sprout interest in the INN catalogue, and we believe that this data set will be used in the future by others, which might concentrate on other aspects.

**Figure 5:**
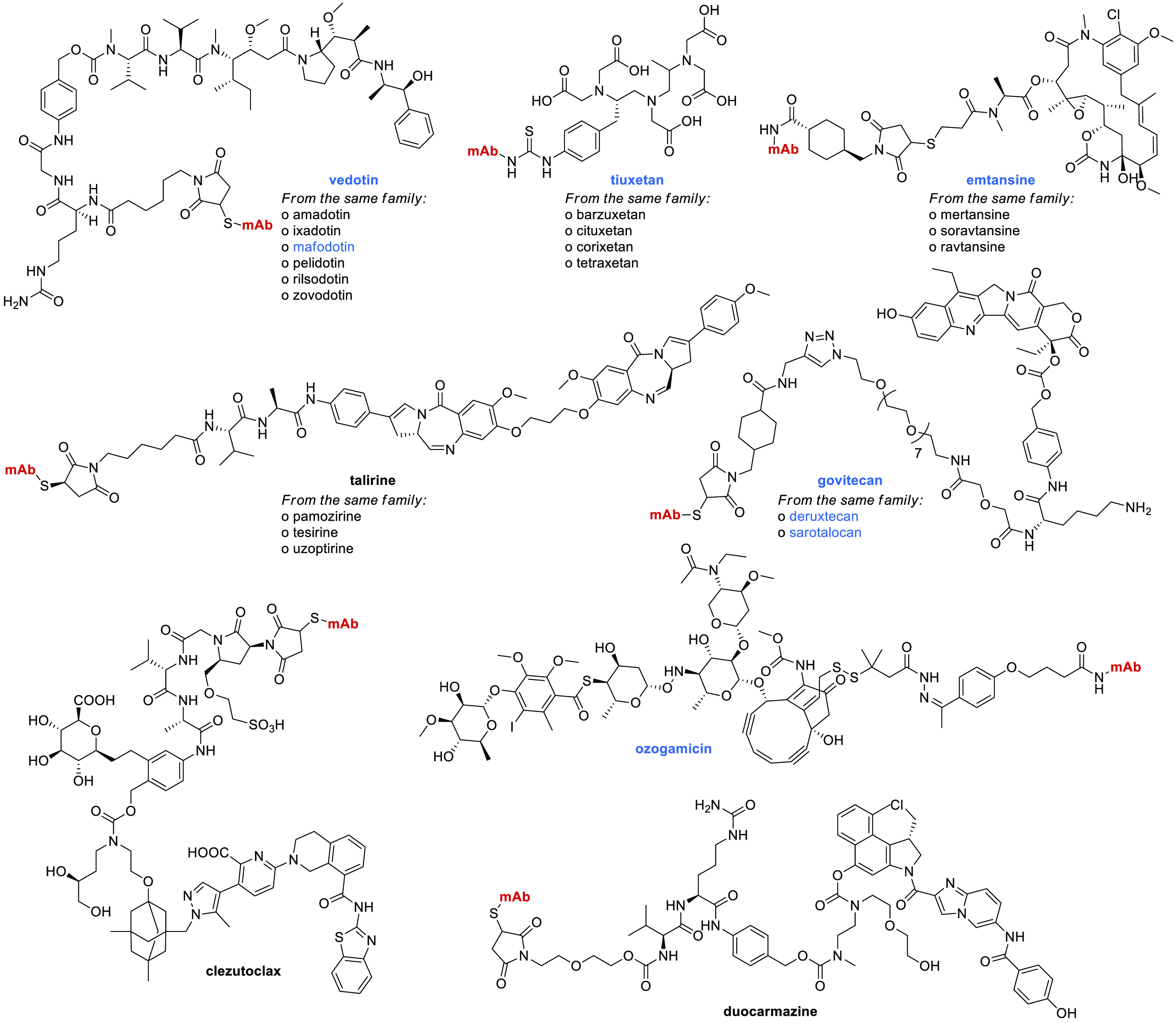
Representative structures of SCEs that are conjugated with mAbs. Payloads that are part of approved ADCs are highlighted in blue.

The decision to concentrate solely on SCEs meant that we excluded mixtures (i.e., plauracin, guaifylline, and latidectin A3+A4) and polymers (*N* = 25, e.g., paclitaxel poliglumex) from our analyses due to the impossibility of assigning them precise physicochemical characteristics, albeit recognizing that these might be of interest to the medicinal chemist. Last, conjugates were also excluded, as this group contained a number of medicines that do not pertain to the medicinal chemistry arena (e.g., mAbs+toxins/peptides, mAbs+radiolabeled isotopes). Yet, the same group also contained 59 antibody-SCE conjugates (antibody-drug conjugates (ADCs)), and we felt that this deserved to be flagged, given that the synthesis and characterization of chemical payloads are becoming an important field in medicinal chemistry and in anticancer therapy.^33,34^ As it can be observed in Figure 5, there are five different families of payloads that are more often used in conjugates: auristatin analogues (e.g., vedotin), maytansinoids (e.g., emtansine), pyrrolobenzodiazepine derivatives (e.g., talirine), irinotecan analogues (e.g., govitecan), and chelating agents (e.g., tiuxetan; to which a radioactive isotope is usually added). Some of the 21 payloads are conjugated with more than one mAb (e.g., labetuzumab govitecan and sacituzumab govitecan). In a similar manner, the same mAb can be conjugated with distinct payloads (e.g., trastuzumab emtansine and trastuzumab deruxtecan, both of which are currently approved).

The different payloads that compose a family (indicated by different INNs with the same stem, such as -*dotin*, -*xetan*, -*tansine*, and -*tecan*) may differ by their linker function or by chemical modifications in the core molecule (although these are not indicated in Figure 5, and a single representative member is depicted). Last, we also found three payloads, namely, ozogamicin, duocarmazine, and clezutoclax, that are unique representatives, not being part of a family of molecules. Eight distinct payloads have been approved so far in 11 ADCs, with vedotin being approved in three different drugs and ozogamicin in two.

### Trends in SCEs Drug Classes Developed

It is probably overambitious to prime the INN catalogue to investigate for diseases that have received the most attention from industry, as the description of drug use present in the database is given early on in development and may not accurately represent the later stages of development (i.e., industries may decide to repurpose a drug for a different disease while in clinical development; see apremilast in Figure 1 as an example). Furthermore, the descriptions are rather vague, and drugs undergo an extension of indications that would not be represented in the initial description.

For this reason, we decided instead to take a “nomenclature” approach and investigated which are the stems (e.g., drug classes) used in at least 10 INNs in the last 20 years. Table 1 lists the most frequent stems found in the 2018 SCEs, their definition as described in the stem book,^9^ and examples of approved drugs for each of them. As it can be observed, a few of these (-*ine*, -*one*, -*fos*-, -*abine*) are traditional and refer to chemical moieties, but most stems nowadays refer to pharmacological targets or to drug use.

**Figure 6:**
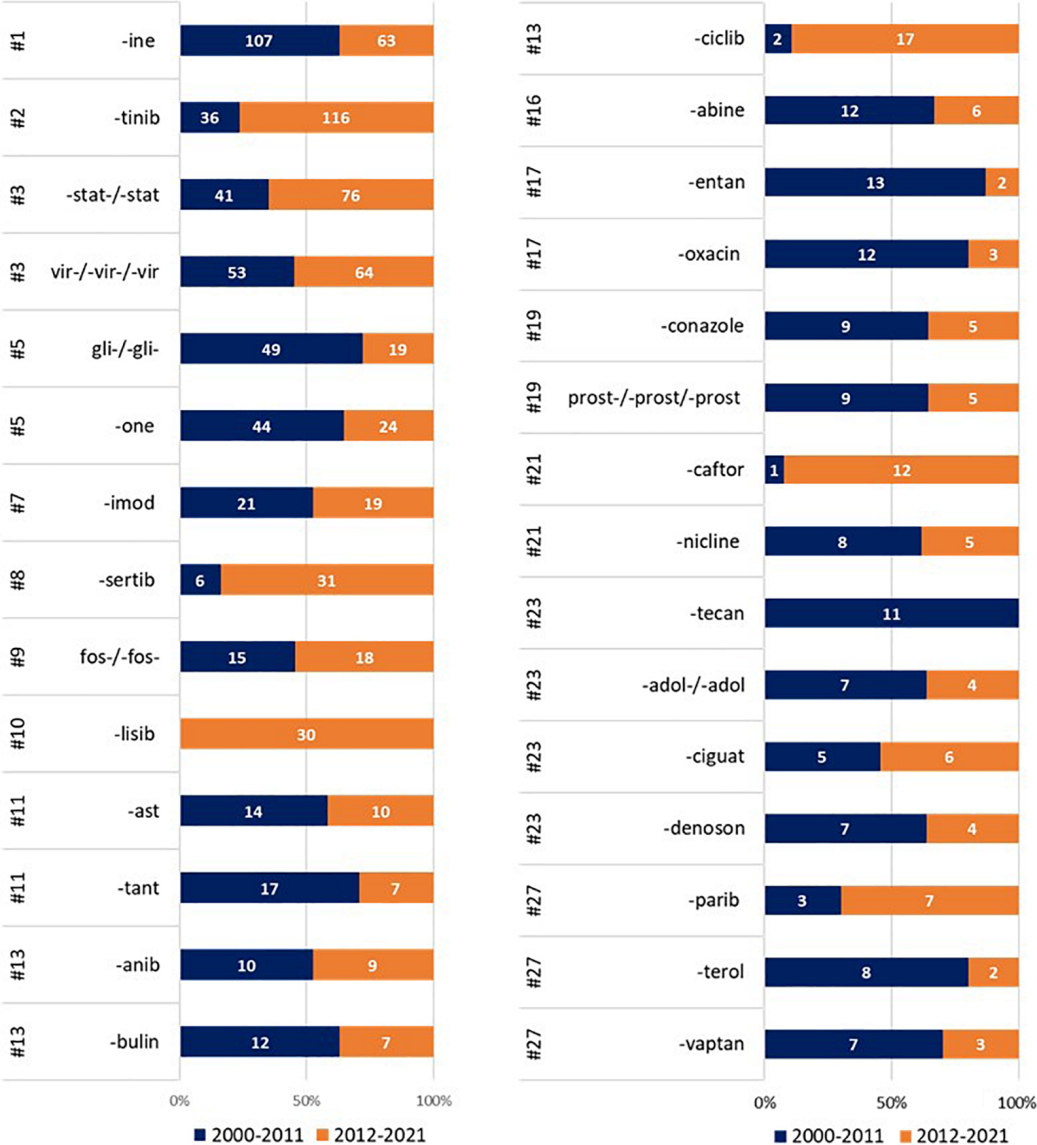
Frequency of the most common stems in period A (blue; 2000–2011: *N* = 1038) and period B (orange; 2012–2021: *N* = 980).

We then analyzed the frequency of these stems in two different periods, subdividing the SCEs in two subsets of a similar size: period A from 2000 to 2011 composed of 1038 SCEs and period B from 2012 to 2021 composed of 980 SCEs (Figure 6). A qualitative analysis reveals some striking changes. First, tyrosine kinase, cyclin-dependent kinase, serine/threonine kinase, and phosphatidylinositol 3-kinase inhibitors have seen a surge of interest among developers in the last 10 years. Almost 20% of all SCEs issued an INN since 2011 belong to one of these four classes, with tyrosine kinase inhibitors accounting for most of them. It is also of interest that, of the 37 serine/threonine kinase inhibitors, none has so far been approved, possibly owing to the recent investment. Possibly an unexpected finding, remaining in the oncology field, is that some chemotherapeutic classes have not gone out of fashion altogether (e.g., -*bulin*, -*abine*), while some others (-*tecan*) have completely lost popularity. Alongside cancer chemotherapeutic agents, a significant drop in INN assignments is also observed for the diabetes field and for some other drug targets (e.g., neurokinin receptors, vasopressin receptors, and endothelin receptors) that were popular in the first decade of the millennium. Of great interest is that a drug class exclusively developed for a single rare disorder (cystic fibrosis) has sprouted sufficient interest to issue 12 names in the last 10 years (-*caftor*). Overall, this analysis points, as expected, to a great dynamism of the pharmaceutical industry, with significant shifts in interest and development, which are bound to shape the drugs eventually approved for the market and the patients cared for.

### Elemental Composition

We then analyzed the elemental composition of the 2018 SCEs in the INN lists. To speed up and automate the analysis process, a python protocol was prepared using a module designed for a chemoinformatic analysis (RDKit).^35^ This protocol made it possible to extract the SMILES structures of INN lists from Pubmed and use them both to calculate chemical descriptors (see below) and to count the number of elements present in each molecule.

**Figure 7:**
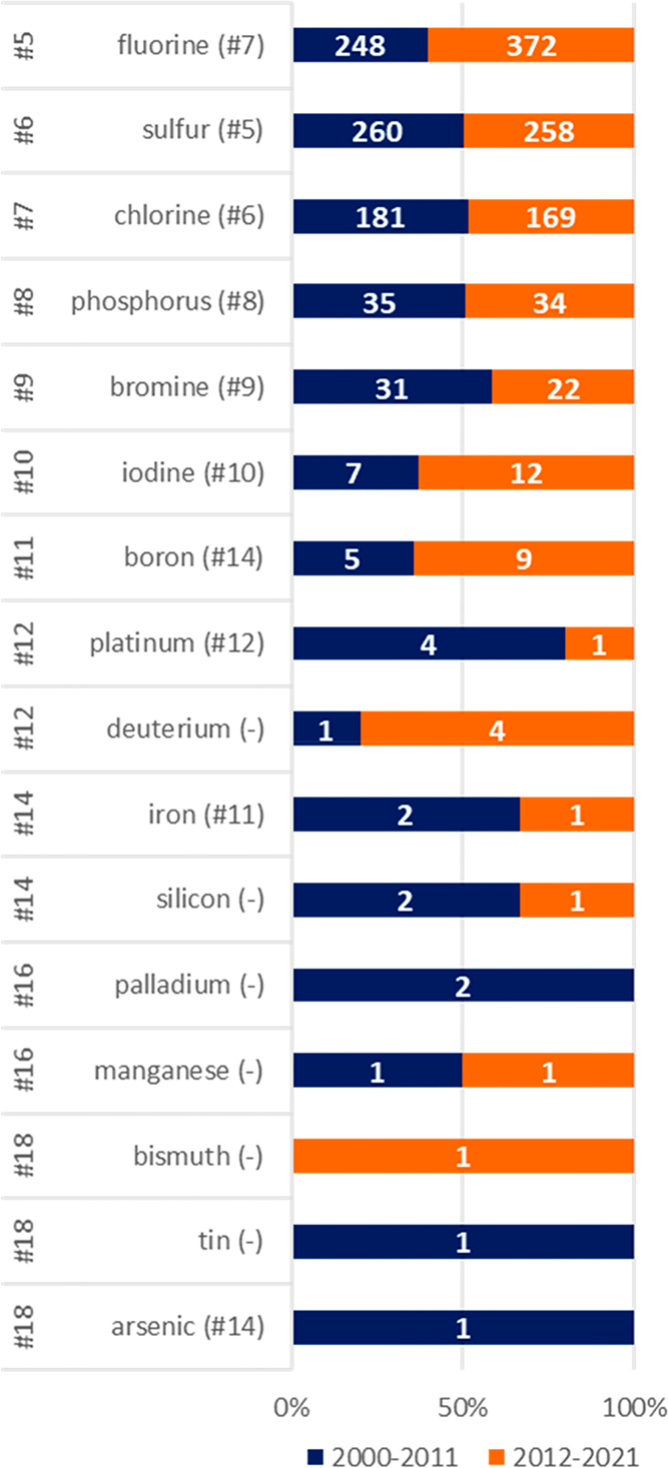
Frequency of elements beyond CHON in SCEs in period A (blue; 2000–2011: *N* = 1038) and period B (orange; 2012–2021: *N* = 980). In the INN lists, four molecules are listed as containing sodium, but a visual inspection revealed that this indicates the salified molecule. These molecules were thereby excluded from the figure. Numbers in parentheses refer to the relative ranking of elements in FDA-approved drugs.^20^ *The occurrence of each element group refers to the number of SCEs containing at least one.

The elemental composition of the 2018 SCEs was compared to the one described in an analysis made on FDA-approved pharmaceuticals in 2014,^20^ and the frequency of elements was compared between periods A and B (Figure 7).

Obviously, carbon, hydrogen, oxygen, and nitrogen (CHON) are present in the vast majority of compounds, as was the case for FDA-approved drugs.^20^ Just after CHON, the INN list presents fluorine in just over 30% of compounds. This shows a change in medicinal chemistry strategies compared to the past, with fluorine taking over from chlorine and sulfur as the most frequent element after CHON.^20^ While this could be somehow expected,^36^ the extent of the use of fluorine might possibly surprise some. Indeed, a recent analysis made on FDA-approved drugs from 2015 to 2020 showed that 26% of drugs displayed fluorine,^37^ already a very high number, but in our analysis we find 40% of drugs in the same time frame (compared to 17% in the first four years of the century) and 55% of fluorinated drugs issued a name in 2020.

No substantial differences were found on the percentage of approved drugs containing chlorine or fluorine, which have remained approximately similar between 2000 and 2020.

Iodine is present in only 19 compounds, a frequency very close to that of boron, which occurs in 14 molecules. The rate of approval of the iodine-containing molecules is lower (11%), while the one of boron-containing molecules is significantly higher (43%) compared to the INN SCE benchmark (20%). Of the 14 boron-containing molecules, nine have been published in period B, demonstrating that the use of this element is increasing over time,^38,39^ although numbers remain small. While most of these molecules present boronic acid (*N* = 8), four contain five-membered boron heterocycles, and two contain six-membered boron heterocycles.

**Figure 8:**
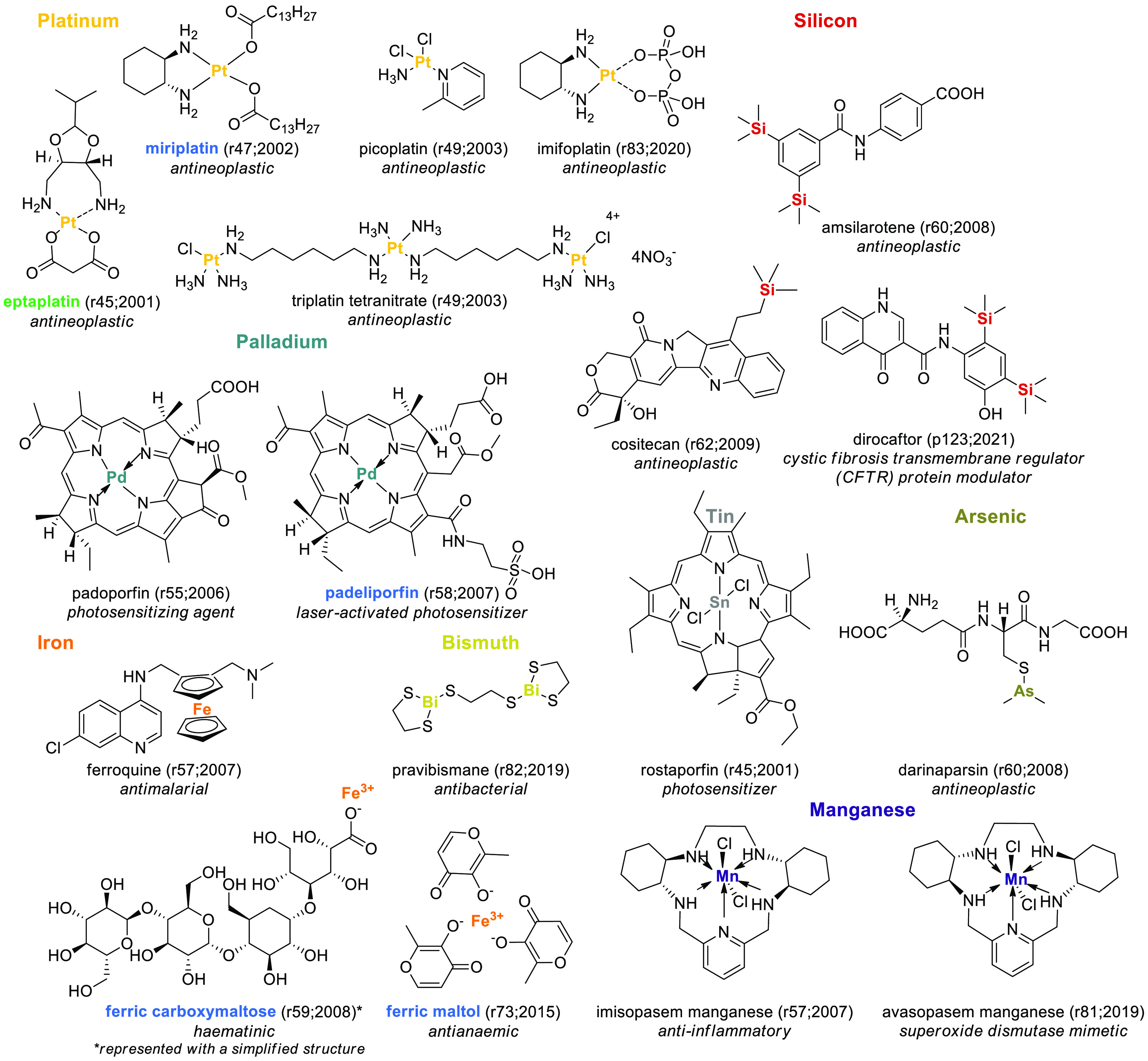
INN molecules containing unconventional elements. EMA-, FDA-, or PMDA-approved SCEs are highlighted in blue, and molecules found to be approved by other agencies are highlighted in green. The numbers in brackets indicate the rINN list and the year in which the molecule was published.

The remaining elements are platinum (*N* = 5), silicon (*N* = 3), iron (*N* = 3), palladium (*N* = 2), manganese (*N* = 2), bismuth (*N* = 1), tin (*N* = 1), and arsenic (*N* = 1), and molecules that contain these uncommon elements are represented in Figure 8.

**Figure 9:**
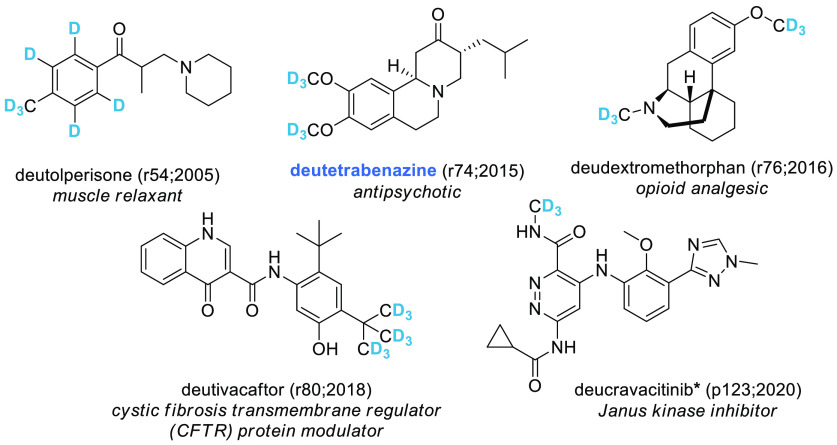
INN deuterated molecules. EMA, FDA, or PMDA approved SCEs are highlighted in blue. The numbers in brackets indicate the rINN list and the year in which the molecule was published. *To avoid a proliferation of stems and definitions, the WHO uses broad categories. Deucravacitinib well exemplifies that this choice is not flawless and might result in grouping molecules with distinct features. Indeed, both the stem (-citinib) and the definition place this drug as a Janus kinase (JAK) inhibitor, although the drug has been designed and developed as a specific inhibitor of TYK2, one of the four protein members of the JAK family (the others being JAK1, JAK2, and JAK3).

Silicon is a proposed carbon isostere that improves the drug-likeness of bioactive compounds,^40,41^ and, similarly, deuterium has gained academic popularity as an isostere of hydrogen.^42−45^ The INN database lists three silicon-containing (Figure 8) and five deuterium-containing molecules (Figure 9). Some of these SCEs are initially developed with silicon or deuterium (e.g., cositecan or deucravacitinib), although it is interesting to note that most deuterium-containing molecules resulted from deuterium switches and that the cystic fibrosis drug ivacaftor has been the object of both a silicon and a deuterium switch (dirocaftor, Figure 8 and deutivacaftor, Figure 9). While for silicon no prefix has been established yet (but the syllable -*si*- is present in two of the three retrieved drugs), deuterated molecules can be easily recognized by the prefix *deu-*/*deut-*, although this is not officially recognized.^46^ The five compounds in Figure 9 represent the only deuterated INNs published so far. However, deuterated molecules are flourishing in the literature. An example of this is provided by the recently published dosimertinib,^47^ a deuterated analogue of osimertinib (r75; 2016). It is likely that this name represents the preferred choice of the drug developer of what the compound should be called, but to our knowledge this is not an official INN and should not be used in the scientific literature, to avoid confusion (indeed, INN rules would suggest that the putative name should be deutosimertinib). Similar confusion is generated by the use of the name donafenib,^48^ which is the deuterated analogue of sorafenib, as this molecule has never been issued an INN (which would putatively be deusorafenib according to the rules). It is important to note that the absence of these two drugs from our analysis is not a drawback of our data set, because these molecules cannot be marketed in Europe or the U.S. unless they reach the WHO for an official INN, which would probably not be the one they have so far used in the scientific literature.

### Functional Groups

**Figure 10:**
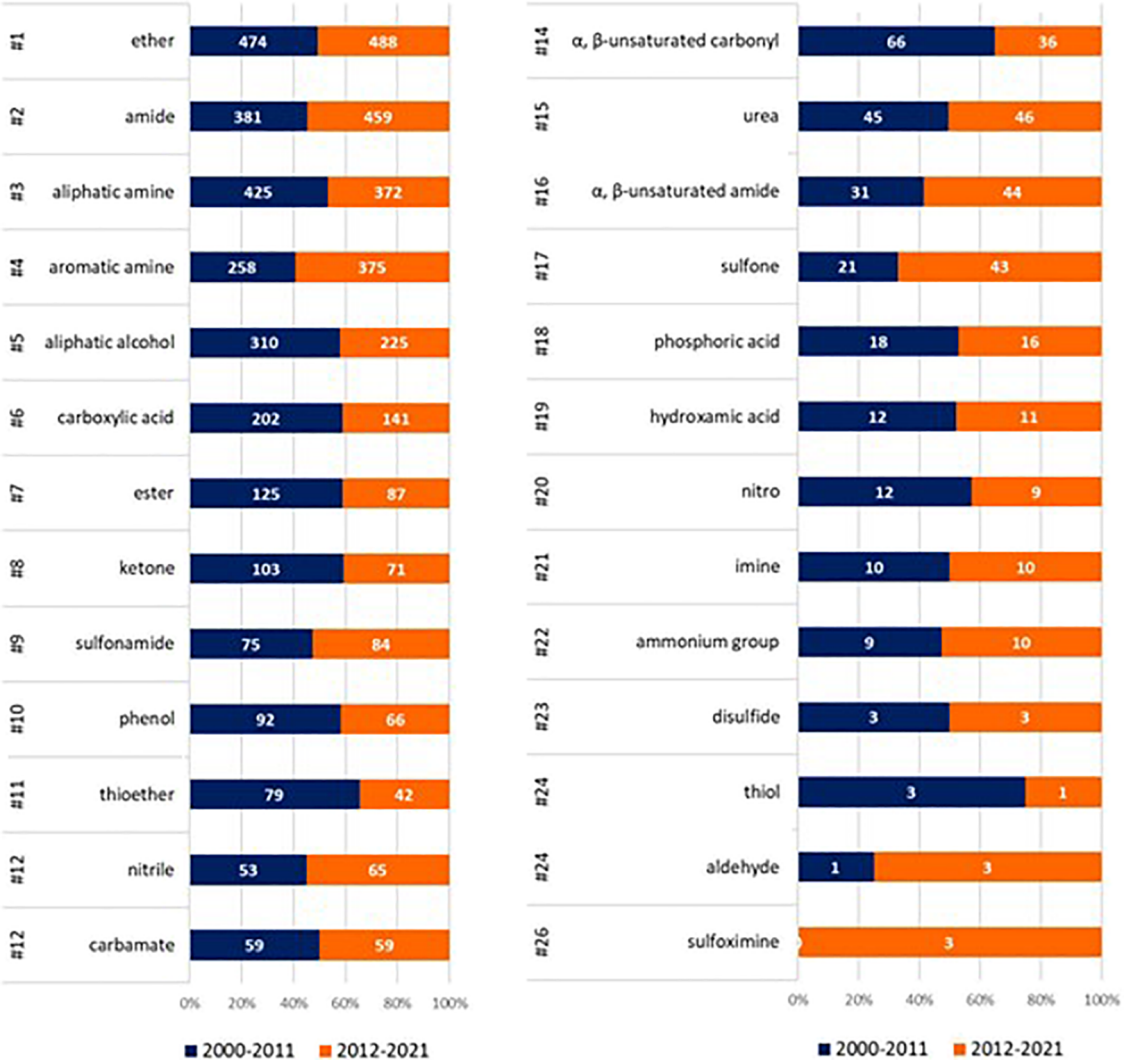
Frequency of selected functional groups in SCEs in period A (blue; 2000–2011: *N* = 1038) and period B (orange; 2012–2021: *N* = 980). *The occurrence of each functional group refers to the number of SCEs containing at least one.

We next evaluated the occurrence of functional groups, taking inspiration from Ertl et al.,^24^ who scanned medicinal chemistry journals, both for the most popular groups to search for and for the in silico approach to employ (we used the SMARTS listed in the Supporting Information using RDKit).^35^ The analysis was then performed subdividing our data set in period A and period B, in order to make a comparison between the two decades (Figure 10).

As shown in Figure 10, the most abundant group is ether (*N* = 962), with approximately half of the INN molecules displaying this group, followed by amide, either linear or lactam (*N* = 840, 42%), aliphatic amine (primary, secondary, and tertiary; *N* = 797, 39%), and aromatic amine (*N* = 633, 31%). The 45% increase in aromatic amines in period B is not surprising considering that kinase inhibitors are particularly rich in this moiety and have significantly increased over time (e.g., *-tinib*, *-sertib*, *-lisib*, and -*ciclib*, see Figure 6).

When looking at the functional groups present in at least 60 molecules, no seismic change between the two periods occurs, although a few trends can be observed. Esters, for example, are usually thought to be groups not privileged in drug design, due to their hydrolytic instability. Despite this, ∼10% of the INN molecules display this group, including prodrugs, even if a trend toward a reduction in period B is observed. Another little change is represented by sulfur-containing functional groups. While the number of SCEs displaying sulfur is the same in the two periods (Figure 7), the use of this element has changed, with a decrease in the use of thioether, a little increase of sulfonamide, and a dramatic rise in sulfone,^17,49^ which is spread over different classes of INNs (e.g., in -*pirdine*, a proposed stem for serotonin receptor antagonists, and in *-sertib* and -*tinib*). While α,β-unsaturated carbonyl groups decrease in period B, α,β-unsaturated amides slightly increase in the same period, despite being well-known structural alerts. Interestingly, their marginal rise is consistent with the surge of covalent kinase inhibitors and follows the approval of afatinib,^50^ ibrutinib,^51^ and osimertinib,^52^ three tyrosine-kinase inhibitors approved as antineoplastic drugs.

**Figure 11:**
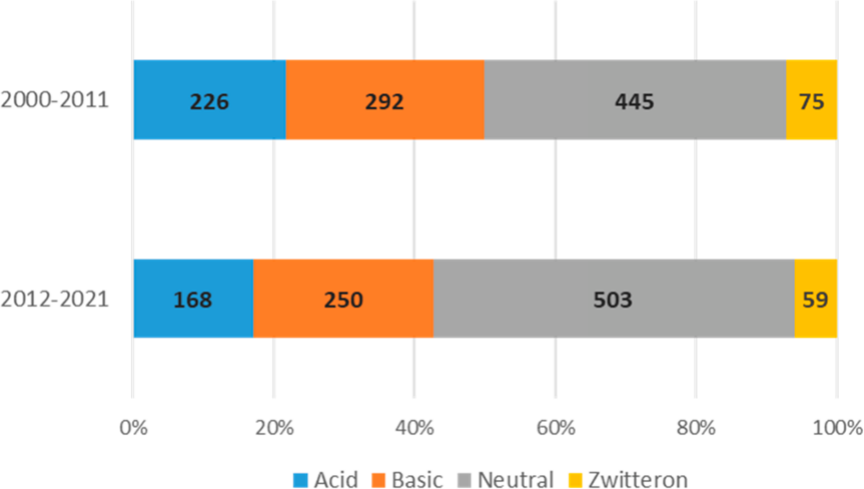
Distribution of neutral, acid, basic, and zwitterionic SCEs in period A (2000–2011: *N* = 1038) and period B (2012–2021: *N* = 980). * To define the acid/base properties of the SCEs, their ionization state at pH 7.4 was considered.

Both carboxylic acids and aliphatic amines have experienced a small decrease in their use over time, and we therefore decided to investigate the fraction of acidic, basic, neutral, and zwitterionic compounds (Figure 11) using an approach similar^53^ to the one described by Charifson et al.^54^ The distribution of the SCEs in the four categories was similar (neutral 47%; basic: 27%; acidic: 19%; zwitterionic: 7%) compared to that described in Charifson et al. with a slight increase of neutral SCEs over time, rising from an average 43% of neutral drugs in period A to 51% in period B.

**Figure 12:**
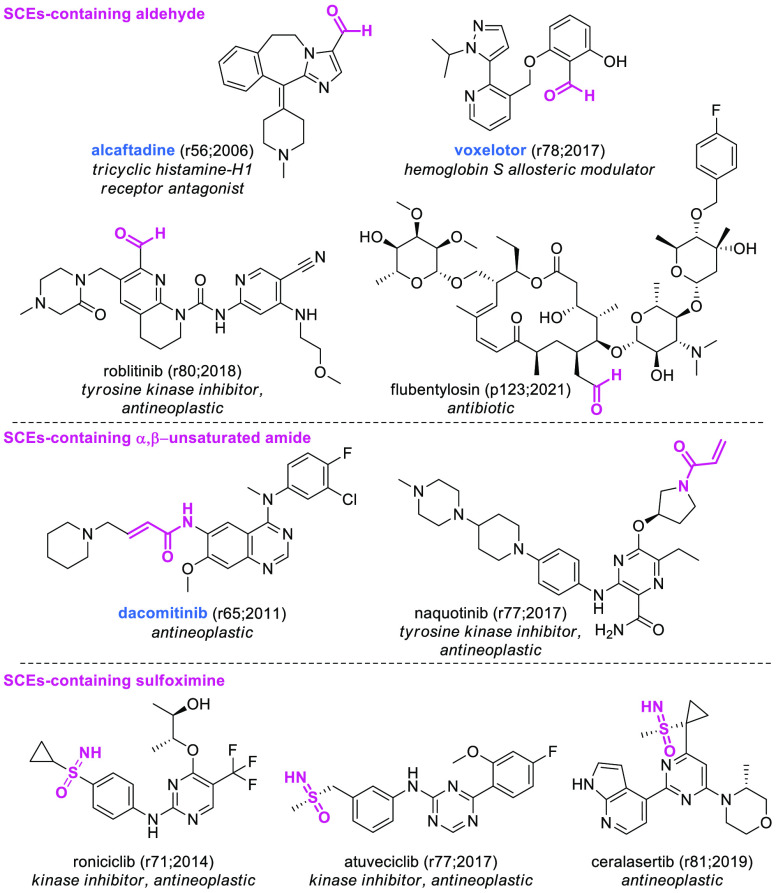
Structures of selected SCEs-containing α,β-unsaturated amide, aldehyde, or sulfoximine. EMA-, FDA-, or PMDA-approved SCEs are highlighted in blue. The numbers in brackets indicate the rINN list and the year in which the molecule was published.

Interestingly, uncommon functional groups are starting to appear: indeed, we found four aldehydes and three sulfoximines (Figure 12). Despite the fact that aldehydes are considered structural alerts in medicinal chemistry^55^ due to their high reactivity toward a vast array of nucleophiles, in our data set two aldehyde-containing SCEs (i.e., alcaftadine in period A, voxelotor in period B) out of four have been approved, confirming that the safety and utility of this functional group should be assessed on a case by case basis in R&D.^56^ Sulfoximine, a neglected functional group in medicinal chemistry, has made its appearance in the lists since 2014. While none of the three SCEs has been approved so far, a recent article pointed at sulfoximine as an emerging group to further expand the toolbox of medicinal chemists.^57^

### The Ring Systems

After having analyzed the functional groups represented in our SCE data set, we investigated the nature of the ring systems by a visual inspection. To do so, we used a slightly modified approach to the original work by Taylor et al.^58^ that considered a ring system as a complete ring or rings formed by removing all terminal and acyclic linking groups.

From a methodological point of view, all rings and fused rings were retained, together with endocyclic bonds and exocyclic carbonyls, sulfonyls, imines, sulfinyls, and thiocarbonyls. Differently from the previous work,^58^ SCEs displaying steroid core substructures^59^ were grouped, while spiro groups were broken into their corresponding rings, as we dedicated a separate analysis to these substructures (see below). We removed macrocycles (number of atoms greater than 11; *N* = 69) as a potentially special case. Similarly to the approach described above, the resulting data set (*N* = 1949) was divided into two subsets: the first (*N* = 994) from 2000 to 2011 (named period A) and the second (*N* = 955) from 2012 to 2021 (named period B). This allowed us to capture the general occurrence of ring systems in the INN list as well as to compare two decades.

Overall, we found 362 distinct ring systems in period A and 419 in period B, with 224 and 257 ring systems used only in a single SCE. This is a small but significant sign that the chemical novelty is increasing and that the accessible chemical space is expanding and also shows the great chemical diversity of INNs. This is further supported by the fact that Taylor et al., when analyzing the 1175 drugs found in the FDA Orange book (which included drugs approved between 1983 and 2012), found 351 distinct ring systems, of which 204 were present only once.

**Figure 13:**
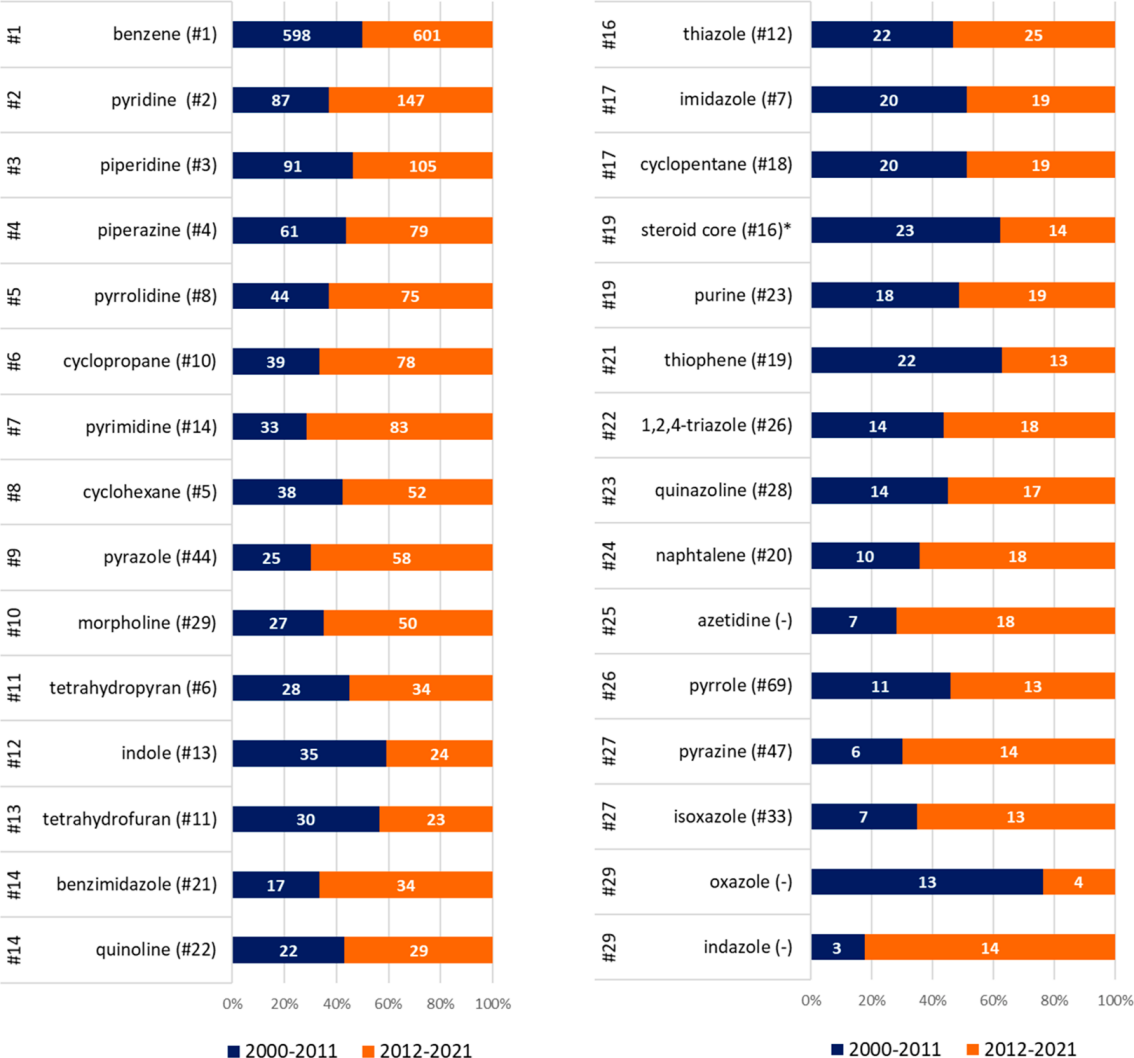
Frequency of selected ring systems in SCEs in period A (blue; 2000–2011: *N* = 994) and period B (orange; 2012–2021: *N* = 955). Numbers in parentheses refer to the relative ranking of ring systems in FDA-approved drugs.^58^*The occurrence of each ring system was determined by counting the number of SCEs containing at least one*. *Steroids were grouped^59^, while in Taylor et al. they were subdivided, considering the different ring systems. With our methodology, steroids would have risen to second place in Taylor et al.^58^

We then compiled the list of the top 30 most frequently used ring systems (Figure 13) and compared it with the one reported by Taylor et al.^59^ The similarity between the two lists confirmed that medicinal chemists still rely on a subset of ring systems that have not changed in the last decades and are in part related to intrinsic properties and synthetic accessibility (e.g., benzene, pyridine, piperidine, cyclohexane). Yet, some substantial changes have occurred: azetidine, oxazole, and indazole are newcomers that were not present in the top 100 ring systems detected by Taylor et al., while pyrazole, pyrazine, and pyrrole have gained prominence. Interestingly, cephalosporins (*N* = 4 in period A; *N* = 2 in period B) and penicillins (*N* = 2 in period A; *N* = 0 in period B) are almost absent in our list, while they are well-represented in the FDA Orange Book, highlighting a progressive decrease of interest in β-lactam antibiotics. Similarly, the phenothiazine core, which is featured in 11 molecules in the FDA-approved drugs, is almost absent in our data set (*N* = 1 in period A; *N* = 1 in period B).

We then compared the ring systems in the two periods. At first glance, it is impressive that most nitrogen-containing aromatic ring systems are more represented in period B. Pyridine, benzimidazole, and pyrazine have doubled or nearly doubled, pyrimidine has almost tripled, and indazole, despite the small numbers, is experiencing a significant increase over time. While we did not investigate this systematically, our impression is that this revolution is largely attributable to the advent of tyrosine kinase inhibitors (-*tinib*, see Figure 6) and, to a lesser extent, to cyclin-dependent kinase inhibitors (-*ciclib*, see Figure 6) and to the fact that they usually contain adenine mimetic scaffolds in their pharmacophore. An increase also occurs in nitrogen-containing aliphatic rings, including azetidine, pyrrolidine, and morphine, as well as in piperidine and piperazine, albeit to a less extent. This is not surprising, as all these substructures are more and more frequently introduced as solubility enhancers. Cyclopropane has significantly increased in period B, and this is mainly related to the recent trend to escape from flatland, as described below. It is interesting to note that, while 1,2,4-triazole shows a steady trend, 1,2,3-triazole, despite being outside the top 30 list, is experiencing a significant increase, thanks to the advent of the click chemistry approach and its exploitation in drug development (*N* = 3 in period A; *N* = 12 in period B).^60^

Overall, when adding up the top 30 rings, we found an ∼25% increase in ring systems (1385 in period A and 1710 in period B). The difference is remarkable, considering that periods A and B display a similar number of molecules (*N* = 994 in period A; *N* = 955 in period B), and we therefore believed that this imbalance deserved in-depth consideration. We wondered what counterbalanced this shortage of ring systems in period A, also in consideration that we observed a small increase in molecular weight (MW) in period B (see below), which per se cannot account for this alone. Once we discarded the possibility that there was a decrease in total phenyl rings in period B (total number of phenyl rings, period A = 897; period B = 880), we then hypothesized that the difference could be related to polycyclic fused ring systems. This was indeed consistent with the fact that, in period A, more steroids (*N* = 23 period A; *N* = 14 period B), irinotecan analogues (-*tecan*; see Figure 6), and taxanes (*-taxel*; see Figure 6) were present, and these, according to the methodology used, were not disconnected. While the polycyclic decrease might partially explain our observation, the numbers are too small to believe that this is the sole explanation. Indeed, it is likely that it is a plethora of small changes that add up to explain a large effect that we observe in ring systems. For example, we also found a difference in the number of exocyclic carbons between period A (*N* = 7149) and period B (*N* = 6125) that, while not being decisive, might partially contribute to fill the observed void.

### Symmetric Compounds

**Figure 14:**
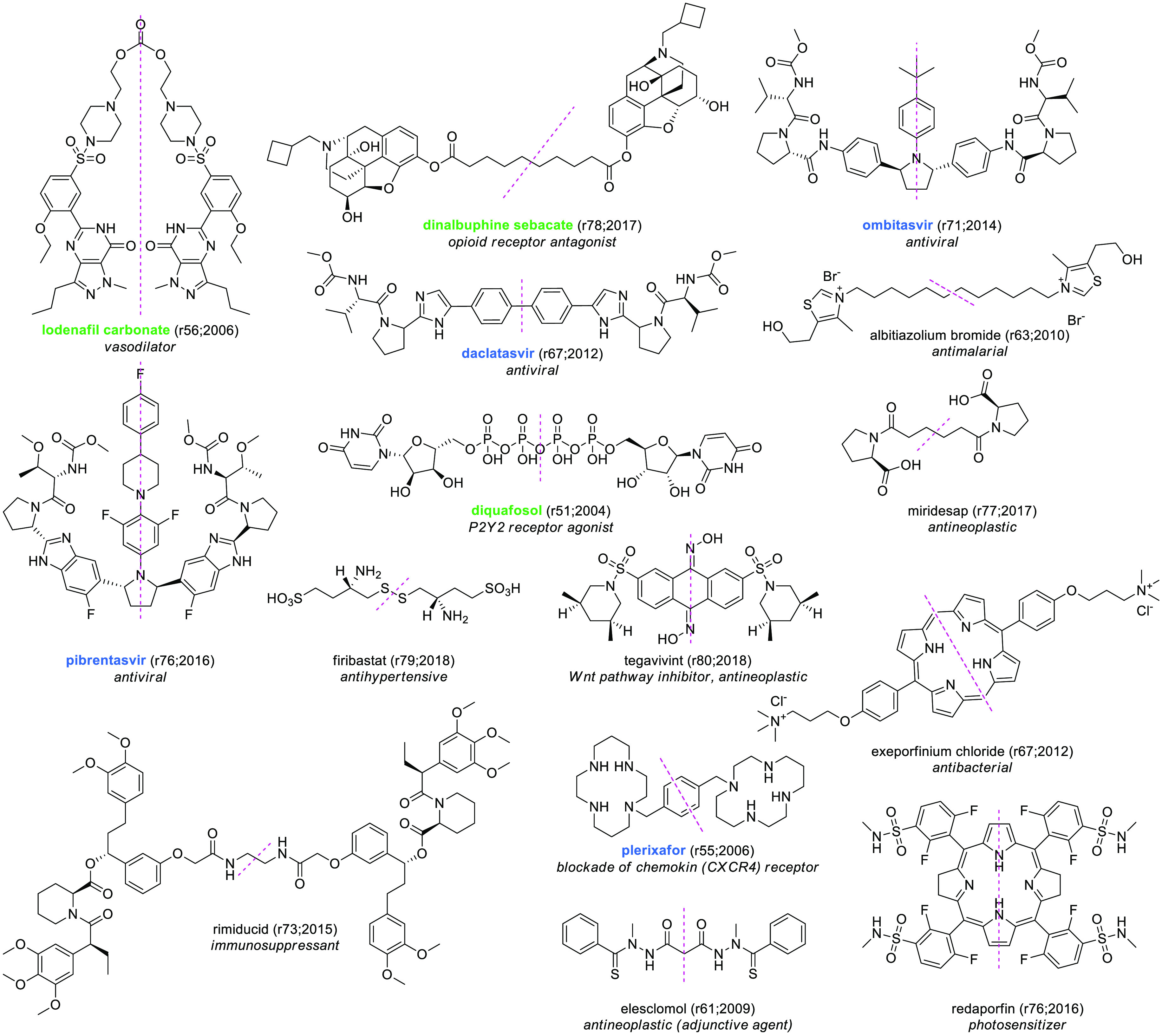
Structures of selected symmetric compounds. EMA-, FDA-, or PMDA-approved SCEs are highlighted in blue, and molecules found to be approved by other agencies are highlighted in green. The numbers in brackets indicate the rINN list and the year in which the molecule was published.

While we were visually inspecting the data set, we were impressed by the abundance of symmetric compounds that were present (*N* = 29, referring to *C*_2_ symmetry). It is interesting that there has been a doubling in the last two decades of these molecules, from 10 between 2000 and 2011 to 19 in the last 10 years. Two compounds are actually prodrugs bearing one inactivating portion and two identical molecules of the active principle (lodenafil carbonate, dinalbuphine sebacate). As expected, most of the remaining symmetric compounds are traditional twin drugs designed to target proteins displaying dimeric structures.^61^ Among them, we found the NS5A inhibitors used as a hepatitis C virus (HCV) treatment (i.e., daclatasvir,^62^ ombitasvir,^63^ pibrentasvir^64^), diquafasol (a P2Y2 antagonist),^65^ firibastat (an aminopeptidase A inhibitor),^66^ and tegavivint (a TBL1 inhibitor).^67^ Similar are those twin drugs intended to bind two identical target proteins (albitiazolium bromide,^68^ miridesap^69^). In this regard, it is interesting to see that the Chemically-Inducible Dimerization (CID) technology^70^ has led to the development of rimiducid,^71^ a tacrolimus analogue that behaves as a protein dimerizer, triggering the homodimerization of Fv-containing drug-binding domains of genetically engineered proteins such as the Caspase 9, Fas intracellular domain, and iCD40 receptor. Molecules where symmetry can be found as a means to complex metals are plerixafor^72^ (zinc) and elesclomol^73^ (copper). Finally, two porfirinic compounds are included (i.e., exeporfinium chloride, redaporfin). Figure 14 shows some selected examples of symmetric molecules.

### Molecular Complexity

It is often believed that medicinal chemistry is becoming more complex to deal with the increasingly challenging drug targets and in line with increased synthetic accessibility. However, chemical complexity remains an elusive concept.^74^ The ranking of compounds mainly depends on what parameters are used to describe complexity, but an unambiguous definition is yet to be defined. While several complexity descriptors have been reported,^74^ what universal parameters should be used as proxies is still a controversial issue. In the literature, a broad range of possibilities is described, from simple topological or physicochemical descriptors to more complex indexes that combine several features into a single score.^75,76^ Since a universally accepted index does not exist and a systematic evaluation of the proposed alternatives is beyond the scope of this review, we decided to consider only some descriptors that are undoubtably linked to complexity, well-aware that further work will be required in this field. It is our hope that this catalogue might be used to prime molecular complexity by others, possibly using a historical approach from 1953.

The first element we investigated was whether molecules are getting larger: bigger and more complex molecules can access greater chemical space, can better complement the three-dimensional target binding site, and can possibly escape from existing Markush formulas. The MW^35^ of INNs is slowly but steadily increasing over time both in the entire data set and in the restricted data set of approved drugs. This can be seen when evaluating the mean, which may be skewed by particularly large drugs, but also when evaluating the median (Figure 16B), which should exclude artifacts given by outliers. Briefly, there is an upward trend seen for all INN compounds (mean 435 in 2000–2004; mean 467 in 2016–2020). Furthermore, the MW of drugs in period B is ∼10% higher compared to those in period A (Figure 16A). Approved INN drugs have a slightly higher MW (mean 478) compared to the overall INN compounds (mean 462) when the mean is compared, which we determined to compare our data with that of the literature. Our data (which necessarily include molecules that will fail along the way) are in contrast with the report that MW significantly decreases at each stage of development, from discovery to market,^76^ but are in line with the hypothesis that a correlation exists between high MW and increased selectivity and reduced attrition rate.^77^

The increment in MW in drugs is not a new trend, as noticed by Ivanenkov et al.,^76^ that saw a strong difference between drugs approved in the past decade compared to drugs approved in the first 50 years of the previous century. This finding is also supported by other reports that show that discovery compounds^78^ as well as marketed drugs and oral drugs^15^ have experienced a consistent time-dependent increase, with a dramatic increment over the past decade.^76^

**Figure 15:**
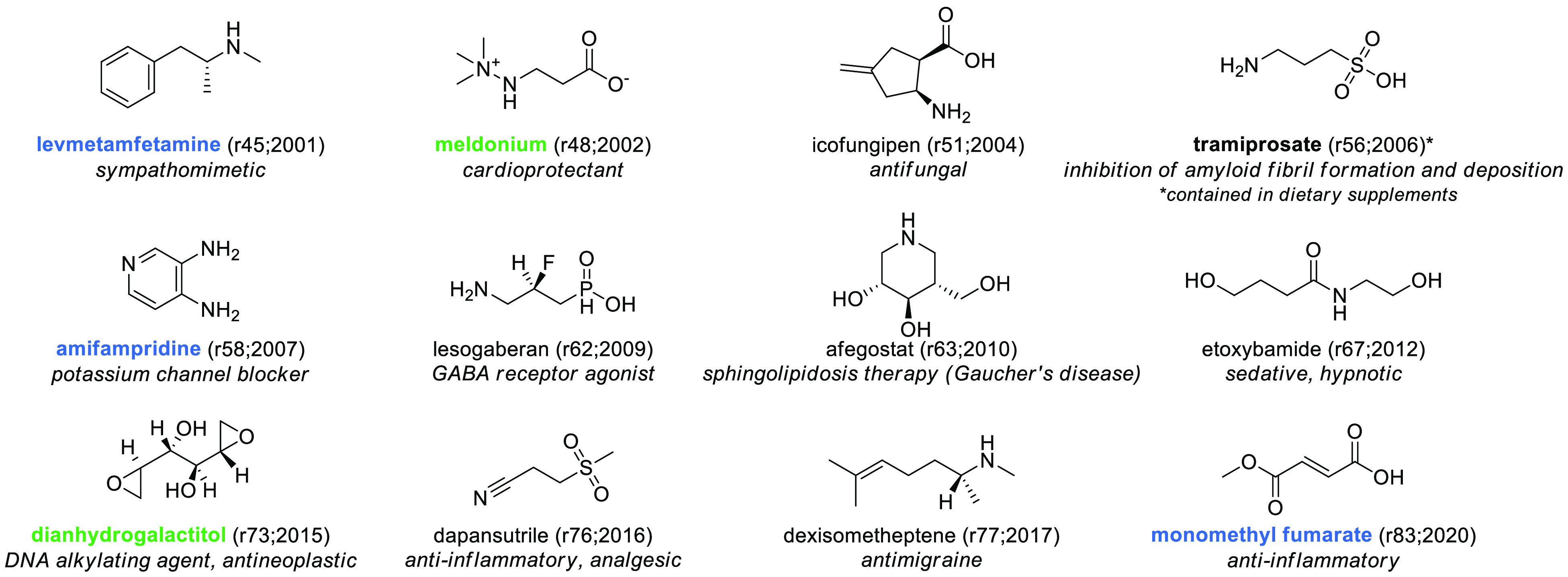
Structures of the 12 SCEs with MW below 150 Da. EMA-, FDA-, or PMDA-approved SCEs are highlighted in blue, and molecules found to be approved by other agencies are highlighted in green. The numbers in brackets indicate the rINN list and the year in which the molecule was published. It is interesting to note that, at times, the INN does not reflect the common name by which a compound is known (which in the case of tramiprosate would be homotaurine).

As recently reported by Raymer et al.,^79^ in spite of the fact that MW is increasing, lead-like drugs (drugs below MW 300) still represent a fruitful area of research and a therapeutic opportunity (2011–2016: 17% of drug approvals), and we find 14% of these molecules in our INN data set. To our great surprise, a substantial number of compounds has an MW below 150, of which five are approved worldwide (three in the main regulated areas we focused on), and one is contained in dietary supplements (Figure 15). Similarly, while 500 is considered the threshold for drug-like compounds, 29% of INN compounds are above this limit, and 11.5% of these are represented by macrocycles (*N* = 69).

In 2009, in their seminal paper Lovering et al. suggested that the medicinal chemistry community should “escape from the flatland” by increasing the fraction sp^3^ (Fsp^3^ = number of sp^3^-hybridized carbons/total carbon count).^80^ The authors proposed Fsp^3^ as an important descriptor of molecular complexity: saturation makes molecules less planar and more structurally complicated, allowing them to access a greater chemical space, without significantly increasing MW. Disruption of molecular planarity is reflected in an increase of aqueous solubility,^80^ target selectivity, and metabolic stability,^81^ and this should in principle improve the clinical success. This prediction is consistent with the finding that Fsp^3^ increases through the five stages of development, going from 0.36 for discovery compounds to 0.47 for drugs on the market.^80^ It has been suggested that a value of Fsp^3^ that is higher than or equal to 0.42 is a suitable benchmark, and 84% of marketed drugs meet this requirement.^82^ In the entire INN data set the mean Fsp^3^ is 0.41,^35^ in accordance with this criterion and with the fact that the INN is usually requested prior to Phase II. Unlike the trend observed by Lovering, Fsp^3^ is identical between approved and not approved drugs.

**Figure 16:**
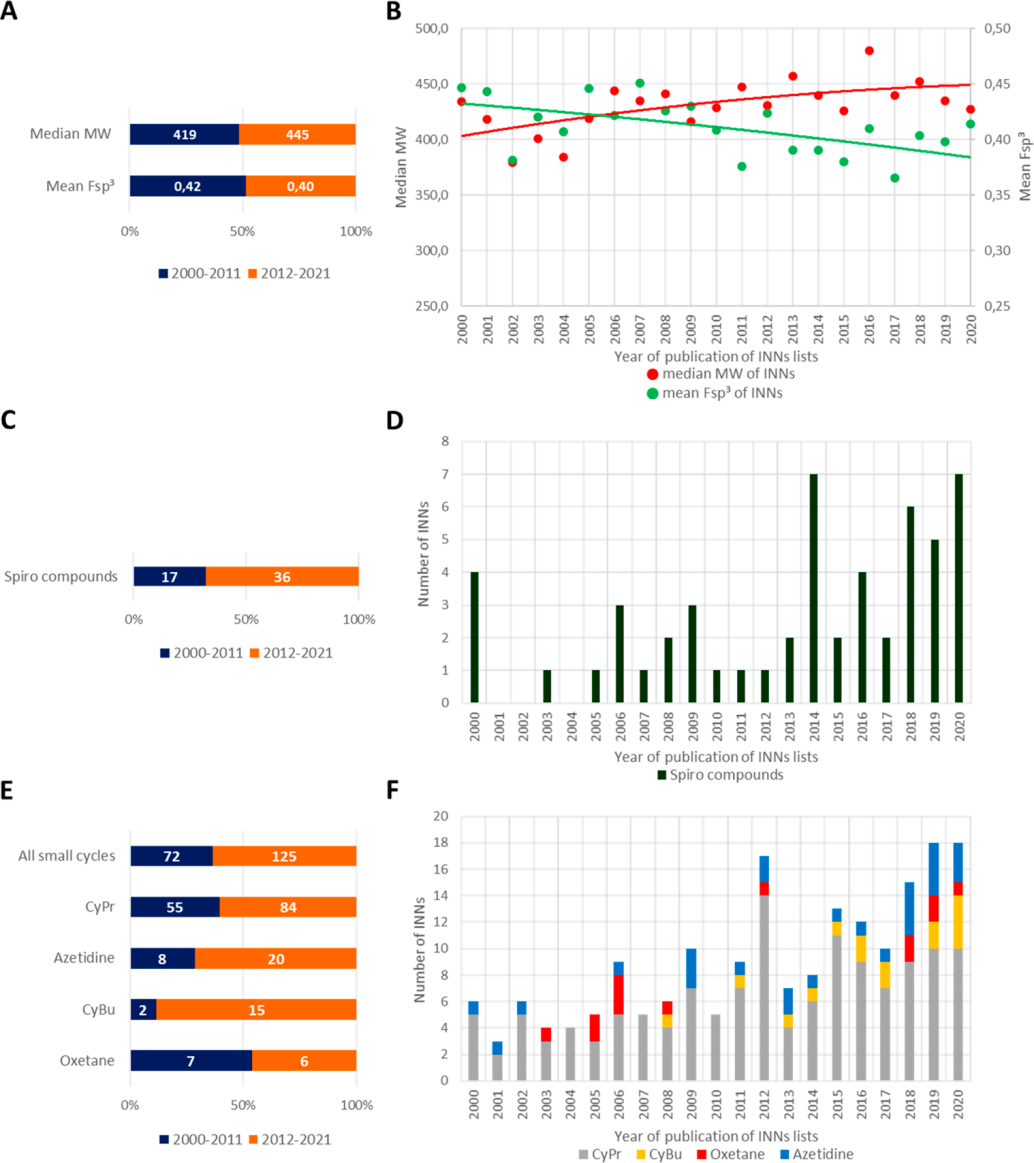
(A) Median MW and mean Fsp^3^ in period A (2000–2011: *N* = 1038) and period B (2012–2021: *N* = 980) and (B) per year; number of spirocycles (C) in periods A and B and (D) per year and number of SCE-containing small cycles (E) in periods A and B and (F) per year. *The occurrence of spiro and small cycles refers to the number of SCEs containing at least one. Benzofused systems were excluded from the count of spiro compounds. Lactones and lactams were excluded from the count of small cycles.

We then evaluated whether medicinal chemistry has escaped from flatland in the last 20 years but found that Fsp^3^ is roughly similar between period A and B (Figure 16A), in line with what was reported by Ivanenkov et al. for launched drugs.^76^ This somehow is a surprising finding, and when attempting to find a trend in the last 20 years, we found a small downward trend (Figure 16B), in analogy to what reported when analyzing discovery compounds published in the *Journal ofMedicinal Chemistry* in the period of 1995–2009.^78^ Despite this, small signs of change toward an enhanced spatial complexity can be observed at the granular level, and we concentrated on chirality, spirocyclic compounds, and small rings.

The chiral nature of drugs has an impact on molecular complexity and correlates with the chance of approval in the process of R&D.^80^ The number of chiral centers^35^ increases through the different steps of clinical stages, reaching the maximum in the approved drugs, where 64% of them have at least one stereocenter. We therefore analyzed the counts of stereocenters in our data set, and we found that 60% of our SCEs have at least one stereocenter, a percentage comparable to that of Phase II compounds,^80^ with a constant trend in the calculated period, 2000–2020. On the contrary, in the same period, the average number of stereocenters per SCE is slightly decreased. We did not undertake a classification of the origin of compounds in our data set (e.g., synthetic, natural, etc.), and therefore we are unable to determine whether a change in the origin has an impact on our findings. Eight SCEs have a number of stereocenters equal to or higher than 20, with a maximum of 35 stereocenters in evernimicin (r44; 2000), followed by pixatimod (r79; 2018) with 29 stereocenters. Chirality is also a regulatory issue, and the FDA-guidelines for the development of chiral active substances published in 1992 prompted the development of pure stereoisomers.^83−85^ It is therefore not surprising that only 10% of all the SCEs containing at least one chiral center are named as a mixture of stereoisomers. Finally, the approval percentage of chiral SCEs is 27%, slightly above the mean of 20%, with no differences among the subset of pure stereoisomers (28%) and mixtures of stereoisomers (26%).

We next focused our attention on spirocyclic motifs that, similarly to quaternary carbon stereocenters, provide an opportunity to project substituents in all three dimensions. Their exploitation in medicinal chemistry has been aided by the recent advances in synthetic strategies that allow access to these substructures.^86^ A significant increase in their use is evident when considering the publications with the keyword “spiro” in the medicinal chemistry field, as done by Hiesinger et al.:^87^ a progressive increase can be found starting from 2000. It is therefore not surprising that, when we analyzed the occurrence over time of spirocyclic motifs^35^ in the INN, more than double spirocycle-containing SCEs have been assigned an INN in period B (Figure 16C) with 62% of them being assigned a name after 2014 (Figure 16D). The impact of these scaffolds is exemplified by two drugs approved in 2020, oliceridine and risdiplam (r76; 2016 and r80; 2018, respectively).

**Figure 17:**
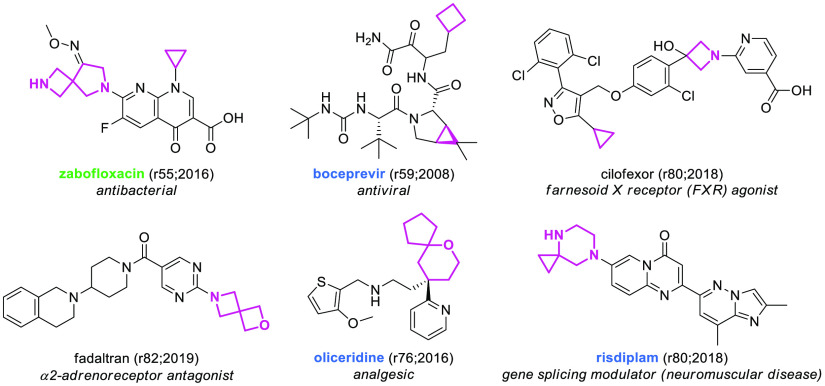
Selected SCEs containing spirocyclic scaffolds and small rings. EMA-, FDA-, or PMDA-approved SCEs are highlighted in blue and molecules found to be approved by other agencies are highlighted in green. The numbers in brackets indicate the rINN list and the year in which the molecule was published.

Besides spirocyclic substructures, aliphatic three- and four-membered rings^35^ contribute to the overall Fsp^3^. While cyclopropanes have been exploited for many years in drug discovery, cyclobutanes, azetidines, and oxetanes have become popular only recently, an uptrend that goes hand-in-hand with the advent of synthetic methods that allow their incorporation. Bauer et al. have recently described the occurrence of these cycles in the patent literature (2009–2019) and have found that cyclopropane is the most-used small ring, followed by cyclobutane, azetidine, and oxetane.^88^ Albeit numbers are small, it is evident that, with the exception of oxetane, all rings have increased over time also in the INN lists, with cyclopropane experiencing the most significant increase (Figure 16F). Interestingly, cyclobutane is not represented in the period of 2000–2007 and makes its entrance in 2008, occurring 15 times since then. Overall, the occurrence of these small cycles in period B compared to period A has almost doubled (Figure 16E). Figure 17 shows some representative examples of molecules bearing spirocyclic scaffolds and small rings.

Overall, therefore, the data from the INN lists show a clear trend in increased MW. Synthetic feasibility has counterbalanced the putative advantages of escaping from flatland, and this is evident when considering the chemical reactions used in current medicinal chemistry, which is overpopulated by amide bond formation, Suzuki-Miyaura coupling, and S_N_Ar reactions.^89^

## Conclusions

A main objective of this review was to disseminate the INN nomenclature schemes and allow readers to recognize the features of a name (stems, infixes, radicals, prefixes, and suffixes) that are important to detect some of the characteristics of the medicine, which may be important for teaching medicinal chemistry and pharmacology, as well as for clinical practice.

A second objective was to evaluate the ∼2000 small molecules that received an INN in the last 20 years, (i) paralleling some of the previous analyses done on different chemical catalogues by others and (ii) comparing the two decades of this century.

Many before us, possibly more skilled, have scrutinized chemical catalogues to describe the essence of pharmaceutical R&D. This has been done on FDA-approved drugs,^14−21^ patents by the pharmaceutical industry,^88,90^ publications by academics,^24^ and drugs under clinical investigation.^91^

Now we propose to use the publications related to the INN, freely accessible on the WHO Web site,^13^ as a new chemical catalogue, that presents different features, possibly advantageous, over other databases. First, compared to patents and publications, these molecules should represent lead compounds that industry has decided to invest in at the clinical level. Second, these publications anticipate the market by approximately four to five years and therefore can foresee trends and changes before the databases on approved medicines. These advantages are obviously counterbalanced by the fact that ∼75% of the molecules in this catalogue will never be approved.

A peculiar finding of our analysis is that medicinal chemistry is largely unchanged, despite the significant modification in drug targets that we describe. Therefore, at first glance, one might be led to believe that medicinal chemistry is largely conservative. Yet, a closer look under the lens shows subtle evolutionary changes that make the baseline constantly drift. Molecular weight is a good example of this, and, while the constant drift might be related to the change in drug targets, it should be noted that this trend follows similar transformations observed since 1910.^76^ Small signs that might pave the path to more significant changes in the future, or might just represent a historical coincidence, also creep up in our analyses. In particular, we identified a cluster of approved boron-containing drugs, a few molecules that incorporate deuterium, and an increased exploitation of small rings (e.g., cyclopropane, cyclobutane, azetidine) and of spirocyclic scaffolds. These small changes are going hand-in-hand with the advent of new synthetic strategies^87−89^ that ease access to such structural features.

Two other results caught our attention: first, among the elements beyond CHON, fluorine is being used above expectations and, in the latest INN publications, is represented in ∼40% of the molecules (with a peak of 55% in the publications from 2020); second, in the last 10 years there has been an increase in the use of N-containing ring systems, with some heterocycles significantly contributing to this (e.g., pyridine, pyrimidine, and pyrazole). Fluorine use had been predicted,^35^ and it is highly likely that the increase in N-containing ring systems is partly linked to the fact that the kinome has become a popular target. We also observed a strong decrease in beta-lactam containing drugs, in steroids, and in phenothiazines, most likely representing a change in therapeutic areas.

While this database will be of great use to scrutinize drugs under development and to inform on trends in medicinal chemistry, it is unlikely to ever allow a determination of the features that confer success to a molecule, as too many variables, alongside chemistry, influence this aspect. In this respect, an acknowledgment of a referenced paper^78^ quotes an anonymous referee that provides an enlightening truth: “*Drugs have to survive multiple hurdles followed by attritional factors including toxicity, clinical safety, efficacy in humans, differentiation, market viability, organizational strategy, regulatory approval and acceptance by payers. It is not a surprise that drug-likeness resists accurate description*.”

## Supporting Information Available

Data set of the INN molecules and list of SMARTS used for searching functional groups (XLSX)

## Abbreviations Used

ADC: antibody-drug conjugate
ANSI: American National Standards Institute
ATC: Anatomical Therapeutic Chemical Classification System
ATMP: advanced therapies
CAS: Chemical Abstract Service
CHON: carbon, hydrogen, oxygen and nitrogen
CID: Chemically-Inducible Dimerization
EGFR: epidermal growth factor receptor
EML: Essential Medicines List
EMA: European Medicines Agency
FDA: Food and Drug Administration
Fsp^3^: fraction sp^3^
HCV: hepatitis C virus
INCI: International Nomenclature Cosmetic Ingredient
INN: international nonproprietary name
INNM: international nonproprietary name modified
ISO: International Standards Organization
IUPAC: International Union of Pure and Applied Chemistry
mAb: monoclonal antibody
MAPK: mitogen-activated protein kinase
MW: molecular weight
NS5B: nonstructural protein 5B
pINN: proposed INN
PMDA: Pharmaceuticals and Medical Devices Agency
rINN: recommended INN
SCE: small chemical entities
U.S.: United States
WHO: World Health Organization

## References

[ref1] a Wikipedia. https://en.wikipedia.org/wiki/List_of_languages_by_total_number_of_speakers (accessed 2020-12-21).

[ref2] World Health Organization. https://apps.who.int/iris/bitstream/handle/10665/86211/WHA3.11_eng.pdf (accessed 2020-12-21).

[ref3] Aronson J. K. “Where name and image meet”–the argument for “adrenaline”. BMJ. 2000, 320, 506–509. 10.1136/bmj.320.7233.506. 10678871 PMC1127537

[ref4] Aronson J. K. Medication errors resulting from the confusion of drug names. Expert Opin. Drug Saf. 2004, 3, 167–172. 10.1517/14740338.3.3.167. 15155145

[ref5] Personal Care Products Council. https://www.personalcarecouncil.org/resources/inci/ (accessed 2020-12-21).

[ref6] Kramer C. V.; Zhang F.; Sinclair D.; Olliaro P. L. Drugs for treating urinary schistosomiasis. Cochrane Database Syst. Rev. 2014, 8, CD000053 10.1002/14651858.CD000053.pub3. PMC4447116 25099517

[ref7] World Health Organization. https://www.who.int/medicines/services/inn/inn_expert_group/en/ (accessed 2020-12-21).

[ref8] World Health Organization. https://www.who.int/medicines/services/inn/publication/en/ (accessed 2020-12-21).

[ref9] Stem Book 2018. https://www.who.int/publications/i/item/who-emp-rht-tsn-2018-1 (accessed 2021-01-27).

[ref10] Radical Book 2015. https://www.who.int/medicines/services/inn/RadicalBook2015.pdf?ua=1 (accessed 2021-01-27).

[ref11] Robertson J. S.; Chui W. K.; Genazzani A. A.; Malan S. F.; López de la Rica Manjavacas A.; Mignot G.; Thorpe R.; Balocco R.; Rizzi M. The INN global nomenclature of biological medicines: A continuous challenge. Biologicals 2019, 60, 15–23. 10.1016/j.biologicals.2019.05.006. 31130314

[ref12] School of International Non-proprietary Names. https://extranet.who.int/soinn/ (accessed 2020-12-21).

[ref13] World Health Organization. https://www.who.int/medicines/publications/druginformation/innlists/en/ (accessed 2020-12-21).

[ref14] Das P.; Delost M. D.; Qureshi M. H.; Smith D. T.; Njardarson J. T. A survey of the structures of US FDA approved combination drugs. J. Med. Chem. 2019, 62, 4265–4311. 10.1021/acs.jmedchem.8b01610. 30444362

[ref15] Shultz M. D. Two decades under the influence of the rule of five and the changing properties of approved oral drugs. J. Med. Chem. 2019, 62, 1701–1714. 10.1021/acs.jmedchem.8b00686. 30212196

[ref16] Delost M. D.; Smith D. T.; Anderson B. J.; Njardarson J. T. From oxiranes to oligomers: Architectures of U.S. FDA approved pharmaceuticals containing oxygen heterocycles. J. Med. Chem. 2018, 61, 10996–11020. 10.1021/acs.jmedchem.8b00876. 30024747

[ref17] Scott K. A.; Njardarson J. T. Analysis of US FDA-approved drugs containing sulfur atoms. Top Curr. Chem. 2018, 376, 5 10.1007/s41061-018-0184-5. 29356979

[ref18] DeGoey D. A.; Chen H. J.; Cox P. B.; Wendt M. D. Beyond the rule of 5: Lessons learned from AbbVie’s drugs and compound collection. J. Med. Chem. 2018, 61, 2636–2651. 10.1021/acs.jmedchem.7b00717. 28926247

[ref19] Vitaku E.; Smith D. T.; Njardarson J. T. Analysis of the structural diversity, substitution patterns, and frequency of nitrogen heterocycles among U.S. FDA approved pharmaceuticals. J. Med. Chem. 2014, 57, 10257–10274. 10.1021/jm501100b. 25255204

[ref20] Smith B. R.; Eastman C. M.; Njardarson J. T. Beyond C, H, O, and N! Analysis of the elemental composition of U.S. FDA approved drug architectures. J. Med. Chem. 2014, 57, 9764–9773. 10.1021/jm501105n. 25255063

[ref21] Ilardi E. A.; Vitaku E.; Njardarson J. T. Data-mining for sulfur and fluorine: an evaluation of pharmaceuticals to reveal opportunities for drug design and discovery. J. Med. Chem. 2014, 57, 2832–2842. 10.1021/jm401375q. 24102067

[ref22] Scott K. A.; Qureshi M. H.; Cox P. B.; Marshall C. M.; Bellaire B. C.; Wilcox M.; Stuart B. A. R.; Njardarson J. T. A Structural analysis of the FDA Green Book-approved veterinary drugs and roles in human medicine. J. Med. Chem. 2020, 63, 15449–15482. 10.1021/acs.jmedchem.0c01502. 33125236

[ref23] Serafini M.; Cargnin S.; Massarotti A.; Pirali T.; Genazzani A. A. Essential medicinal chemistry of essential medicines. J. Med. Chem. 2020, 63, 10170–10187. 10.1021/acs.jmedchem.0c00415. 32352778 PMC8007110

[ref24] Ertl P.; Altmann E.; McKenna J. The most common functional groups in bioactive molecules and how their popularity has evolved over time. J. Med. Chem. 2020, 63, 8408–8418. 10.1021/acs.jmedchem.0c00754. 32663408

[ref25] Brown D. G.; Boström J. Where do recent small molecule clinical development candidates come from?. J. Med. Chem. 2018, 61, 9442–9468. 10.1021/acs.jmedchem.8b00675. 29920198

[ref26] These substances have been labeled with an asterisk in the Supporting Information database, since their proposed INNs were submitted by WHO several years before their publication in the rINN list.

[ref27] On the one hand, the category of biologicals includes proteins, including those in which some amino acid residues have been modified, low molecular weight heparins, purified hormones, and small peptides. On the other hand, when a short peptide (up to four-five amino acids) was present in an otherwise SCE (e.g., vintafolide, r69;2013), this substance was inserted in the SCE category. Note that the number of mAbs is slightly overestimated, as often, in the same rINN list, the applicant applied for the mAb itself together with a second application for the relative conjugate (30 examples in the analyzed lists). Pegylation, indicated as either the prefix “peg” or the suffix “pegol”, has not been considered in the classification of the substances. RNA/DNA-based therapies broadly comprise antisense, siRNA, and mRNA-based therapies. Advanced therapies comprise gene and cell therapies, oncolytic viruses, or bacteria. The category of veterinary drugs has been compiled following the indication reported in the proposed INN volumes, and not on chemical or biological structures, but it must be recognized that also drugs not specifically categorized as veterinary during the INN application process may be then developed for veterinary use. Surprisingly, the lists also include a few sunscreens, possibly because these are not considered cosmetic ingredients for the U.S. legislation.

[ref28] Belzupacap sarotalocan is described in list p122 as “A modified human papillomavirus (HPV) type 16-derived empty nanoparticle, 55 nm in diameter conjugated to approximately 200 molecules of a phthalocyanine-based photosensitizer (sarotalocan group). Each nanoparticle is comprised of 72 capsomeres, made of 5 molecules of modified viral capsid protein L1 [P78 > R, T176 > N, D273 > T, N285 > T, S288 > N, T353 > P, T389 > S] and one molecule of viral capsid protein L2”.

[ref29] FDA-approved drugs. https://www.accessdata.fda.gov/scripts/cder/daf/ (accessed 2020-09-02).

[ref30] EMA EPARs for human and veterinary medicines. https://www.ema.europa.eu/en/medicines/download-medicine-data (accessed 2020-09-02).

[ref31] PMDA-approved drugs. https://www.pmda.go.jp/english/review-services/reviews/approved-information/drugs/0002.html (accessed 2020-09-02).

[ref32] aadisinsight.springer.com (accessed 2021-01-25).

[ref33] Bargh J. D.; Isidro-Llobet A.; Parker J. S.; Spring D. R. Cleavable linkers in antibody-drug conjugates. Chem. Soc. Rev. 2019, 48, 4361–4374. 10.1039/C8CS00676H. 31294429

[ref34] Casi G.; Neri D. Antibody-Drug Conjugates and small molecule-drug conjugates: opportunities and challenges for the development of selective anticancer cytotoxic agents. J. Med. Chem. 2015, 58, 8751–61. 10.1021/acs.jmedchem.5b00457. 26079148

[ref35] RDKit, 2020.09.1 version: Open-Source Cheminformatics Software. https://www.rdkit.org. (accessed 2020-08-07).

[ref36] Meanwell N. A. Fluorine and fluorinated motifs in the design and application of bioisosteres for drug design. J. Med. Chem. 2018, 61, 5822–5880. 10.1021/acs.jmedchem.7b01788. 29400967

[ref37] Bhutani P.; Joshi G.; Raja N.; Bachhav N.; Rajanna P. K.; Bhutani H.; Paul A. T.; Kumar R. U.S. FDA approved drugs from 2015–June 2020: a perspective. J. Med. Chem. 2021, 64, 2339–2381. 10.1021/acs.jmedchem.0c01786. 33617716

[ref38] Song S.; Gao P.; Sun L.; Kang D.; Kongsted J.; Poongavanam V.; Zhan P.; Liu X. Recent developments in the medicinal chemistry of single boron atom-containing compounds. Acta Pharm. Sin. B [Online early access]. 2021 10.1016/j.apsb.2021.01.010 PMC8546671 34729302

[ref39] Fernandes G. F. S.; Denny W. A.; Dos Santos J. L. Boron in drug design: Recent advances in the development of new therapeutic agents. Eur. J. Med. Chem. 2019, 179, 791–804. 10.1016/j.ejmech.2019.06.092. 31288128

[ref40] Ramesh R.; Reddy D. S. Quest for novel chemical entities through incorporation of silicon in drug scaffolds. J. Med. Chem. 2018, 61, 3779–3798. 10.1021/acs.jmedchem.7b00718. 29039662

[ref41] Kleemiss F.; Justies A.; Duvinage D.; Watermann P.; Ehrke E.; Sugimoto K.; Fugel M.; Malaspina L. A.; Dittmer A.; Kleemiss T.; Puylaert P.; King N. R.; Staubitz A.; Tzschentke T. M.; Dringen R.; Grabowsky S.; Beckmann J. Sila-ibuprofen. J. Med. Chem. 2020, 63, 12614–12622. 10.1021/acs.jmedchem.0c00813. 32931274

[ref42] Pirali T.; Serafini M.; Cargnin S.; Genazzani A. A. Applications of deuterium in medicinal chemistry. J. Med. Chem. 2019, 62, 5276–5297. 10.1021/acs.jmedchem.8b01808. 30640460

[ref43] Cargnin S.; Serafini M.; Pirali T. A primer of deuterium in drug design. Future Med. Chem. 2019, 11, 2039–2042. 10.4155/fmc-2019-0183. 31538524

[ref44] Liu J. F.; Harbeson S. L.; Brummel C. L.; Tung R.; Silverman R.; Doller D. A decade of deuteration in medicinal chemistry. Annu. Rep. Med. Chem. 2017, 50, 519–542. 10.1016/bs.armc.2017.08.010.

[ref45] Gant T. G. Using deuterium in drug discovery: leaving the label in the drug. J. Med. Chem. 2014, 57, 3595–3611. 10.1021/jm4007998. 24294889

[ref46] A hydrogen-bearing molecule can potentially exist in several deuterated forms. For example, the d_9_-analogue of ivacaftor has been named deutivacaftor, but a d_18_-analogue was also reported; therefore, it is possible that, in the future, a prefix alongside the suffix *deu*- will be added to identify the presence of more than one deuterated analogue of a hydrogenated molecule.

[ref47] Meng Y.; Yu B.; Huang H.; Peng Y.; Li E.; Yao Y.; Song C.; Yu W.; Zhu K.; Wang K.; Yi D.; Du J.; Chang J. Discovery of dosimertinib, a highly potent, selective, and orally efficacious deuterated EGFR targeting clinical candidate for the treatment of non-small-cell lung cancer. J. Med. Chem. 2021, 64, 925–937. 10.1021/acs.jmedchem.0c02005. 33459024

[ref48] Bi F.; Qin S.; Gu S.; Bai Y.; Chen Z.; Wang Z.; Ying J.; Lu Y.; Meng Z.; Pan H.; Yang P.; Zhang H.; Chen X.; Xu A.; Liu X.; Meng Q.; Wu L.; Chen F. Donafenib versus sorafenib as first-line therapy in advanced hepatocellular carcinoma: An open-label, randomized, multicenter phase II/III trial. J. Clin. Oncol. 2020, 38, 4506–4506. 10.1200/JCO.2020.38.15_suppl.4506. PMC8445562 34185551

[ref49] Feng M.; Tang B.; Liang S. H.; Jiang X. Sulfur containing scaffolds in drugs: Synthesis and application in medicinal chemistry. Curr. Top. Med. Chem. 2016, 16, 1200–1216. 10.2174/1568026615666150915111741. 26369815 PMC4877035

[ref50] Santos E. S.; Hart L. Advanced squamous cell carcinoma of the lung: current treatment approaches and the role of afatinib. OncoTargets Ther. 2020, 13, 9305–9321. 10.2147/OTT.S250446. PMC7519820 33061419

[ref51] Brown J. R. PCI-32765, the first BTK (Bruton’s tyrosine kinase) inhibitor in clinical trials. Curr. Hematol. Malig. Rep. 2013, 8, 1–6. 10.1007/s11899-012-0147-9. 23296407 PMC3584329

[ref52] Yver A. Osimertinib (AZD9291)—a science-driven, collaborative approach to rapid drug design and development. Ann. Oncol. 2016, 27, 1165–1170. 10.1093/annonc/mdw129. 26961148

[ref53] To calculate p*K*_a_ values, the p*K*_a_ plugin of Marvin’s cxcalc program was used. Marvin 20.19.0, ChemAxon: https://www.chemaxon.com (accessed 2020-08-07).

[ref54] Charifson P. S.; Walters W. P. Acidic and basic drugs in medicinal chemistry: A perspective. J. Med. Chem. 2014, 57, 9701–9717. 10.1021/jm501000a. 25180901

[ref55] Alves V.; Muratov E.; Capuzzi S.; Politi R.; Low Y.; Braga R.; Zakharov A. V.; Sedykh A.; Mokshyna E.; Farag S.; Andrade C.; Kuz’min V.; Fourches D.; Tropsha A. Alarms about structural alerts. Green Chem. 2016, 18, 4348–4360. 10.1039/C6GC01492E. 28503093 PMC5423727

[ref56] Gampe C.; Verma V. A. Curse or cure? A perspective on the developability of aldehydes as active pharmaceutical ingredients. J. Med. Chem. 2020, 63, 14357–14381. 10.1021/acs.jmedchem.0c01177. 32916044

[ref57] Mäder P.; Kattner L. Sulfoximines as rising stars in modern drug discovery? Current status and perspective on an emerging functional group in medicinal chemistry. J. Med. Chem. 2020, 63, 14243–14275. 10.1021/acs.jmedchem.0c00960. 32870008

[ref58] Taylor R. D.; MacCoss M.; Lawson A. D. G. Rings in drugs. J. Med. Chem. 2014, 57, 5845–5859. 10.1021/jm4017625. 24471928

[ref59] Steroid core substructures that were grouped are the following: hexadecahydro-1*H*-cyclopenta[*a*]phenanthrene; 2,3,6,7,8,9,10,11,12,13,14,15,16,17-tetradecahydro-1*H*-cyclopenta[*a*]phenanthrene; 7,8,9,11,12,13,14,15,16,17-decahydro-6*H*-cyclopenta[*a*]phenanthrene; 6,7,8,9,10,11,12,13,14,15,16,17-dodecahydro-1*H*-cyclopenta[*a*]phenanthren-3(2*H*)-one; 6,7,8,11,12,13,14,15,16,17-decahydro-1*H*-cyclopenta[*a*]phenanthren-3(2*H*)-one; 6,7,8,9,10,11,12,13,14,15,16,17-dodecahydro-3*H*-cyclopenta[*a*]phenanthren-3-one.

[ref60] Serafini M.; Pirali T.; Tron G. C. Click 1,2,3-triazoles in drug discovery and development: from the flask to the clinic? Adv. Heterocycl. Chem. 2020, [Online early access]. 10.1016/bs.aihch.2020.10.001.

[ref61] Contreras J.-M.; Sippl W. Homo and Heterodimer Ligands the Twin Drug Approach. In The Practice of Medicinal Chemistry*,* 3rd ed.; Wermuth C. G., Ed.; Elsevier, 2008; pp 380–414.

[ref62] Gamal N.; Gitto S.; Andreone P. Efficacy and safety of daclatasvir in hepatitis C: an overview. J. Clin. Transl. Hepatol. 2016, 4, 336–344. 10.14218/JCTH.2016.00038. 28097103 PMC5225154

[ref63] Flisiak R.; Flisiak-Jackiewicz M. Ombitasvir and paritaprevir boosted with ritonavir and combined with dasabuvir for chronic hepatitis C. Expert Rev. Gastroenterol. Hepatol. 2017, 11, 559–567. 10.1080/17474124.2017.1309284. 28317409

[ref64] Liu X.; Hu P. Efficacy and safety of glecaprevir/pibrentasvir in patients with chronic HCV infection. J. Clin. Transl. Hepatol. 2021, 9, 125–132. 10.14218/JCTH.2020.00078. 33604263 PMC7868694

[ref65] von Kügelgen I. Pharmacology of P2Y receptors. Brain Res. Bull. 2019, 151, 12–24. 10.1016/j.brainresbull.2019.03.010. 30922852

[ref66] Marc Y.; Boitard S. E.; Balavoine F.; Azizi M.; Llorens-Cortes C. Targeting brain aminopeptidase A: a new strategy for the treatment of hypertension and heart failure. Can. J. Cardiol. 2020, 36, 721–731. 10.1016/j.cjca.2020.03.005. 32389345

[ref67] Nomura M.; Rainusso N.; Lee Y. C.; Dawson B.; Coarfa C.; Han R.; Larson J. L.; Shuck R.; Kurenbekova L.; Yustein J. T. Tegavivint and the β-catenin/ALDH axis in chemotherapy-resistant and metastatic osteosarcoma. J. Natl. Cancer Inst. 2019, 111, 1216–1227. 10.1093/jnci/djz026. 30793158 PMC6855956

[ref68] Wein S.; Maynadier M.; Bordat Y.; Perez J.; Maheshwari S.; Bette-Bobillo P.; Tran Van Ba C.; Penarete-Vargas D.; Fraisse L.; Cerdan R.; Vial H. Transport and pharmacodynamics of albitiazolium, an antimalarial drug candidate. Br. J. Pharmacol. 2012, 166, 2263–2276. 10.1111/j.1476-5381.2012.01966.x. 22471905 PMC3437492

[ref69] Pepys M. B. The pentraxins 1975–2018: serendipity, diagnostics and drugs. Front. Immunol. 2018, 9, 2382 10.3389/fimmu.2018.02382. 30459761 PMC6232782

[ref70] Voß S.; Klewer L.; Wu Y.-W. Chemically induced dimerization: reversible and spatiotemporal control of protein function in cells. Curr. Opin. Chem. Biol. 2015, 28, 194–201. 10.1016/j.cbpa.2015.09.003. 26431673

[ref71] Sonpavde G.; McMannis J. D.; Bai Y.; Seethammagari M. R.; Bull J. M. C.; Hawkins V.; Dancsak T. K.; Lapteva N.; Levitt J. M.; Moseley A.; Spencer D. M.; Slawin K. M. Phase I trial of antigen-targeted autologous dendritic cell-based vaccine with in vivo activation of inducible CD40 for advanced prostate cancer. Cancer Immunol. Immunother. 2017, 66, 1345–1357. 10.1007/s00262-017-2027-6. 28608115 PMC11029714

[ref72] Gerlach L. O.; Jakobsen J. S.; Jensen K. P.; Rosenkilde M. R.; Skerlj R. T.; Ryde U.; Bridger G. J.; Schwartz T. W. Metal ion enhanced binding of AMD3100 to Asp262 in the CXCR4 receptor. Biochemistry 2003, 42, 710–717. 10.1021/bi0264770. 12534283

[ref73] Vo N. H.; Xia Z.; Hanko J.; Yun T.; Bloom S.; Shen J.; Koya K.; Sun L.; Chen S. Synthesis, crystallographic characterization and electrochemical property of a copper(II) complex of the anticancer agent elesclomol. J. Inorg. Biochem. 2014, 130, 69–73. 10.1016/j.jinorgbio.2013.10.005. 24176921

[ref74] Méndez-Lucio O.; Medina-Franco J. L. The many roles of molecular complexity in drug discovery. Drug Discovery Today 2017, 22, 120–126. 10.1016/j.drudis.2016.08.009. 27575998

[ref75] Böttcher T. An additive definition of molecular complexity. J. Chem. Inf. Model. 2016, 56, 462–470. 10.1021/acs.jcim.5b00723. 26857537

[ref76] Ivanenkov Y. A.; Zagribelnyy B. A.; Aladinskiy V. A. Are we opening the door to a new era of medicinal chemistry or being collapsed to a chemical singularity?. J. Med. Chem. 2019, 62, 10026–10043. 10.1021/acs.jmedchem.9b00004. 31188596

[ref77] Hopkins A. L.; Mason J. S.; Overington J. P. Can we rationally design promiscuous drugs?. Curr. Opin. Struct. Biol. 2006, 16, 127–136. 10.1016/j.sbi.2006.01.013. 16442279

[ref78] Walters W. P.; Green J.; Weiss J. R.; Murcko M. A. What do medicinal chemists actually make? A 50-year retrospective. J. Med. Chem. 2011, 54, 6405–6416. 10.1021/jm200504p. 21755928

[ref79] Raymer B.; Bhattacharya S. K. Lead-like drugs: a perspective. J. Med. Chem. 2018, 61, 10375–10384. 10.1021/acs.jmedchem.8b00407. 30052440

[ref80] Lovering F.; Bikker J.; Humblet C. Escape from flatland: increasing saturation as an approach to improving clinical success. J. Med. Chem. 2009, 52, 6752–6756. 10.1021/jm901241e. 19827778

[ref81] Lovering F. Escape from Flatland 2: complexity and promiscuity. MedChemComm 2013, 4, 515–519. 10.1039/c2md20347b.

[ref82] Kombo D. C.; Tallapragada K.; Jain R.; Chewning J.; Mazurov A. A.; Speake J. D.; Hauser T. A.; Toler S. 3D molecular descriptors important for clinical success. J. Chem. Inf. Model. 2013, 53, 327–342. 10.1021/ci300445e. 23244494

[ref83] FDA’s policy statement for the development of new stereoisomeric drugs. Chirality 1992, 4, 338–340 10.1002/chir.530040513. 1354468

[ref84] U.S. Food & Drug Administration. https://www.fda.gov/regulatory-information/search-fda-guidance-documents/development-new-stereoisomeric-drugs (accessed 2021-01-25).

[ref85] Murakami H. From racemates to single enantiomers - Chiral synthetic drugs over the last 20 years. Top. Curr. Chem. 2006, 269, 273–299. 10.1007/128_2006_072. 23605355

[ref86] Talele T. T. Opportunities for tapping into three-dimensional chemical space through a quaternary carbon. J. Med. Chem. 2020, 63, 13291–13315. 10.1021/acs.jmedchem.0c00829. 32805118

[ref87] Hiesinger K.; Dar’in D.; Proschak E.; Krasavin M. Spirocyclic scaffolds in medicinal chemistry. J. Med. Chem. 2021, 64, 150–183. 10.1021/acs.jmedchem.0c01473. 33381970

[ref88] Bauer M. R.; Di Fruscia P.; Lucas S. C. C.; Michaelides I. N.; Nelson J. E.; Storer R. I.; Whitehurst B. C. Put a ring on it: application of small aliphatic rings in medicinal chemistry. RSC Med. Chem. 2021, [Online early access]. 10.1039/D0MD00370K. PMC8083977 33937776

[ref89] Brown D. G.; Boström J. Analysis of past and present synthetic methodologies on medicinal chemistry: where have all the new reactions gone?. J. Med. Chem. 2016, 59, 4443–4458. 10.1021/acs.jmedchem.5b01409. 26571338

[ref90] Leeson P. D.; Springthorpe B. The influence of drug-like concepts on decision-making in medicinal chemistry. Nat. Rev. Drug Discovery 2007, 6, 881–890. 10.1038/nrd2445. 17971784

[ref91] Lajiness M. S.; Vieth M.; Erickson J. Molecular properties that influence oral drug-like behavior. Curr. Opin. Drug Discovery Devel. 2004, 7, 470–477. 15338956

